# Supplementary material for: Two-optical-cycle pulses from nanophotonic two-color soliton compression
Source: Light Sci Appl. 2026 Feb 6;15:107. doi: 10.1038/s41377-026-02187-8 (PMC12881584; doi:10.1038/s41377-026-02187-8)
Supplement: Supplementary file 1 — Supplementary Information for Two-optical-cycle pulses from nanophotonic two-color soliton compression. [file 41377_2026_2187_MOESM1_ESM.pdf]

Supplementary Information for “Two-optical-cycle pulses from nanophotonic two-color soliton compression”

Robert M. Gray[1], Ryoto Sekine[1], Maximilian Shen[1], Thomas Zacharias[1], James Williams[1], Selina Zhou[1], Rahul Chawlani[1], Luis Ledezma[1], Nicolas Englebert[1], and Alireza Marandi[1†]

[1]Department of Electrical Engineering, California Institute of Technology, Pasadena, CA 91125, USA

[†]Email: marandi@caltech.edu

Contents

1 Experimental Details **3**

1.1 Experimental Setup 3

1.2 FROG Processing 6

1.3 FROG Measurement Limitations 8

1.4 Device Design and Characterization 12

2 Theoretical Analysis **15**

2.1 Soliton Solutions to Coupled Wave Equations 15

2.2 Numerical Soliton Solutions 23

2.3 Lagrangian Analysis 27

2.4 Pulse Compression Simulations 30

2.5 Theoretically-Informed Design Rules 31

2.6 Relationship to Soliton Number 35

2.7 Full Simulation and Mapping to Experiment 37

2.8 Extension to Longer Pump Pulses 44

2.9 Pulse Synthesis 44

1 Experimental Details

1.1 Experimental Setup

The experimental setup is shown in Fig. S1a. The input pulses at 2090 nm (red lines) which drive the compression are taken from the output of a free-space degenerate optical parametric oscillator based on periodically poled lithium niobate. It is pumped by a commercial mode-locked laser at 1045 nm (Menlo Orange, blue lines) which delivers 103-fs pulses at a repetition rate of 250 MHz. The OPO output consists of nearly transform-limited, 35-fs, sech-shaped pulses centered at 2090 nm. It is then passed through a long-pass filter, beam expander, and variable ND wheel before being sent to the chip. This results in a small amount of anomalous pre-chirp on the pulses. To avoid additionally dispersing the pulses, they are coupled in and out of the thin-film lithium niobate (TFLN) chip using reflective objectives. A pair of magnetic mirrors placed before the chip may also be used to re-direct the 2-μm beam to our home-built frequency-resolved optical gating (FROG) system, described in the following section.

The output of the TFLN device (purple lines) may be directed along one of two measurement paths. Along one path (dashed line), the output is coupled to a multimode fiber using a reflective collimator for direct spectrum measurements using an optical spectrum analyzer (OSA). Along the second path, the output is guided using plano-metallic mirrors to our FROG. A magnetic mounted beamsplitter at the FROG input can be used to switch between second-harmonic generation (SHG) auto-FROG and sum-frequency generation (SFG) X-FROG geometries. The solid lines in the figure illustrate the X-FROG geometry. An off-axis parabolic mirror is used for collimation into the nonlinear crystal, which is a 50-μm-long β-barium borate (BBO) crystal cut for type-I non-collinear phase matching of SFG between 1045 nm and 1300 nm. The crystal is mounted with rotational and translational degrees of freedom to optimize the SHG or SFG signal at the FROG output.

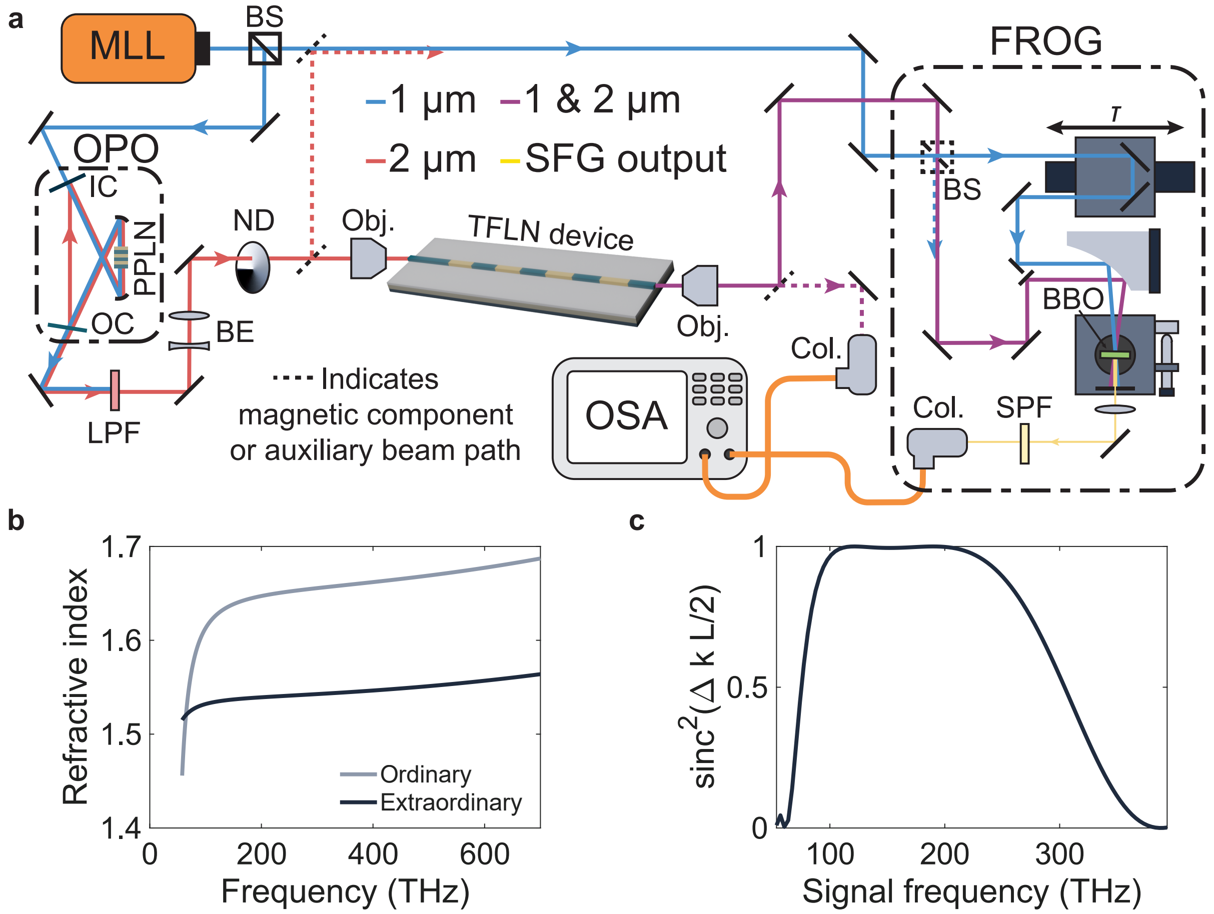

Figure S1: **Experimental setup for measuring two-color soliton pulse compression.** **a**, Setup for generation and measurement of compressed pulses. **b**, Refractive index of BBO crystal used in FROG setup. **c**, Phase-matching curve for sum-frequency generation between the 1045-nm gate pulse and the compressed signal pulse. MLL, mode-locked laser; BS, beam-splitter; OPO, optical parametric oscillator; IC, input coupler; OC, output coupler; PPLN, periodically poled lithium niobate; Obj., reflective objective; TFLN, thin-film lithium niobate; Col., reflective collimator; FROG, frequency-resolved optical gating; BBO, β-barium borate; LPF, long-pass filter; SPF, short-pass filter

The FROG output is sent through a pinhole to filter the residual input beams and then passed through a collimating lens. Finally, a reflective collimator is used to couple the beam to a multi-mode fiber before detection on a spectrometer. The 1045-nm mode-locked laser and 2090-nm OPO output are measured using the auto-FROG geometry. The low-power chip output, however, is measured using an X-FROG, gated by a portion the 1045-nm MLL output. In this X-FROG configuration, we additionally use a short-pass filter (Thorlabs FESH0800), which has a pass band of 500-789 nm, to filter any scattered light at 1045 nm from the high-power gate beam. This limits the bandwidth of our X-FROG measurement to wavelengths between 950 nm and 3220 nm coming from the chip, although this well-captures the most crucial spectral window for the two-color soliton pulse compression.

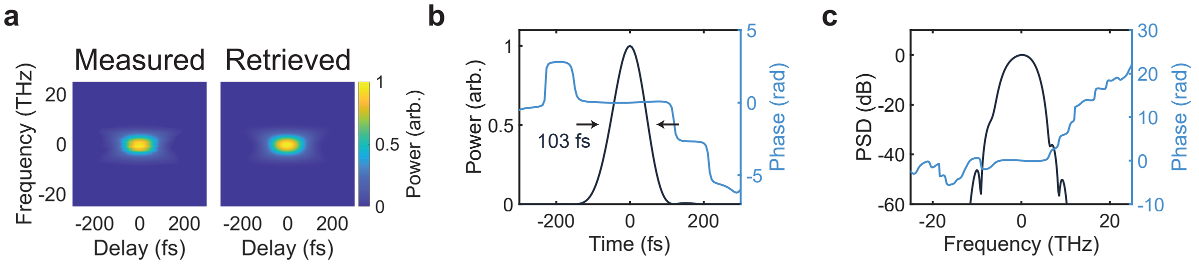

Figure S2: **Gate pulse characterization.** **a**, Measured (left) and retrieved (right) SHG FROG spectrograms for the gate pulse used in the measurement. **b**, Corresponding retrieved temporal profile and **c**, spectrum. FROG error = 0.0012.

To verify the feasibility of using our FROG to measure the broadband pulses coming out of the chip, we simulate the phase-matching bandwidth of the BBO crystal. The refractive index of BBO, shown in Fig. S1b for both the ordinary and extraordinary rays, is found using the Sellmeier equation of ref. [1]. We then compute the phase-matching curve, assuming a fixed gate pulse at 1045 nm and a variable signal frequency. We consider a crystal angle of 23.74 degrees, optimized for phase-matched SFG of 1045 nm and 1550 nm. In practice, this requires rotation of the crystal by -1.26 degrees with respect to normal based on the calculated cut angle of the

crystal of 25 degrees. We further consider an input beam angle of 8.5 degrees for both beams, based on a 1.2” separation between the beams prior to the parabolic mirror and the 4” focal length of the mirror.

The resulting plot of $\mathrm{sinc}(\Delta kL/2)$, where L = 50 μm is the length of the crystal and Δk is the calculated phase mismatch, is shown in Fig. S1c. Here, we observe that the 3-dB bandwidth extends from about 75 THz to 305 THz, well-capturing both the fundamental and second-harmonic frequencies at 143.5 THz and 287 THz, respectively. However, in combination with the short-pass filter response, we believe the observed decrease in phase matching at high frequencies to be responsible for the slight under-estimation of the second-harmonic power in our FROG reconstruction as compared to the measured spectrum on the OSA.

An SHG FROG characterization of 1045-nm gate pulses may be seen in Figure S2. Figure S2a shows the measured and retrieved FROG spectrograms. Good qualitative agreement is observed as well as a reasonable FROG error of 0.0012. The retrieved temporal profile and spectrum are shown in Figs. S2b and S2c, respectively.

1.2 FROG Processing

The output of an SFG X-FROG is an intensity spectrogram[2], which approximately takes the form:

$$I_{FROG}(\omega,\tau) = \left| \int_{-\infty}^{\infty} E_S(t)E_G(t-\tau)e^{-i\omega t}dt \right|^2. \quad \text{(S1)}$$

Here, $E_S(t)$ and $E_G(t)$ are the electric field amplitudes of the signal and gate pulses, respectively. In our FROG system (Fig. S1a), τ is imposed through a mechanical delay stage on the gate arm. Moving from an SFG X-FROG to an SHG auto-FROG merely requires setting $E_G(t)$ equal to $E_S(t)$.

For accurate measurement of broadband few- or single-cycle pulses, several additional con-

siderations come into play. These can include the phase-matching bandwidth, the frequency response of the spectrometer, the frequency dependence of the nonlinear susceptibility, and the frequency dependence of the nonlinear coupling coefficient[2]. As discussed above, the phase-matching bandwidth is sufficiently broad to include the dominant fundamental and second harmonic components well within the 3 dB bandwidth, so we do not factor it into our post processing. We additionally do not consider the effects of the frequency dependence of the nonlinear susceptibility nor the spectrometer response, beyond the built-in amplitude correction of the Thorlabs CCS200 instrument used in the X-FROG measurement.

With these considerations in mind, our post-processing consists of the following. We first perform background subtraction by measuring an empty trace (where no signal is present on the spectrometer) and subtracting it from all other measured traces. After subtraction, we set all values less than 0 to be 0, as a measured value of less than 0 in the spectrogram would be unphysical. Next, we perform thresholding of any content that is more than 20 dB below the maximum measured intensity. Following thresholding, we correct for the frequency dependence of the nonlinear response and by rescaling the spectrogram by a factor of ω^{-2}. Furthermore, the spectrometer measures along a uniform grid in wavelength which is to be resampled to a uniform grid in frequency, requiring an additional ω^{-2} correction to the power per mode. For fair direct comparison between FROG spectra, simulated spectra, and directly measured spectra in our experiment, this ω^{-2} correction is also applied to all measured spectra. Lastly, we normalize the spectrogram to contain only values between 0 and 1 and perform interpolation using the MATLAB interp1 function onto a Fourier grid for processing. For the X-FROG traces, a Fourier grid size of 1024 is used with a total time window length of 2500 fs. A grid size of 1024 is also used for reconstructing the 2-µm pump and 1-µm gate pulses.

Our FROG algorithm uses a slightly modified version of the open-source code provided by Wyatt and Byrnes[3,4]. It employs the principal component generalized projections algorithm de-

veloped by Kane[5,6]. The definition of the Fourier grid and overall performance of the algorithm has been tested against the suggestions made by DeLong, et al.[7]. We run the algorithm for 1000 iterations, although convergence is typically observed within the first 100 iterations. Typical errors for our FROG retrievals are on the order of 10^{-3}, consistent with reported guidelines.

1.3 FROG Measurement Limitations

Our current experimental apparatus has several limitations which prohibit our successfully measuring shorter pulses than what is shown in the main text, although current measurements and simulations (see Section 2.7) suggest that shorter pulse widths should be observed at slightly higher pulse energies. The current limitations are experimentally demonstrated through the measurements presented in Fig. S3.

The measured and retrieved spectrograms for an input pulse energy of 5 pJ are shown in Fig. S3a. Both retrievals show good agreement with the measured spectrogram as well as a low error of 0.0044. Furthermore, good agreement is seen between the retrieved spectrum and measured spectrum on the OSA (Fig. S3b), besides the expected discrepancies due to the FROG bandwidth and presence of higher-order modes as noted in the main text. For retrieval 1, ultrashort pulses of duration 10 fs and 13 fs are observed at both the fundamental and second harmonic, respectively, as shown in Figs. S3c and S3e. The spectral shape and short durations are both consistent with expectations based on simulation (see Section 2.7). However, despite exhibiting similar error, retrieval 2 recovers a different profile for the fundamental wave, depicted in Fig. S3d. Instead of a single, 10-fs pulse, we observe a double-humped pulse of total duration 22 fs. The second harmonic for retrieval 2 is not shown, as it is nearly identical to that of retrieval 1. As can be seen through the corresponding spectra in Figs. S3f and S3g, the predominant discrepancy between the two retrievals is attributable to the relative phase between the high-frequency and low-frequency components of the fundamental wave. When in phase, a single

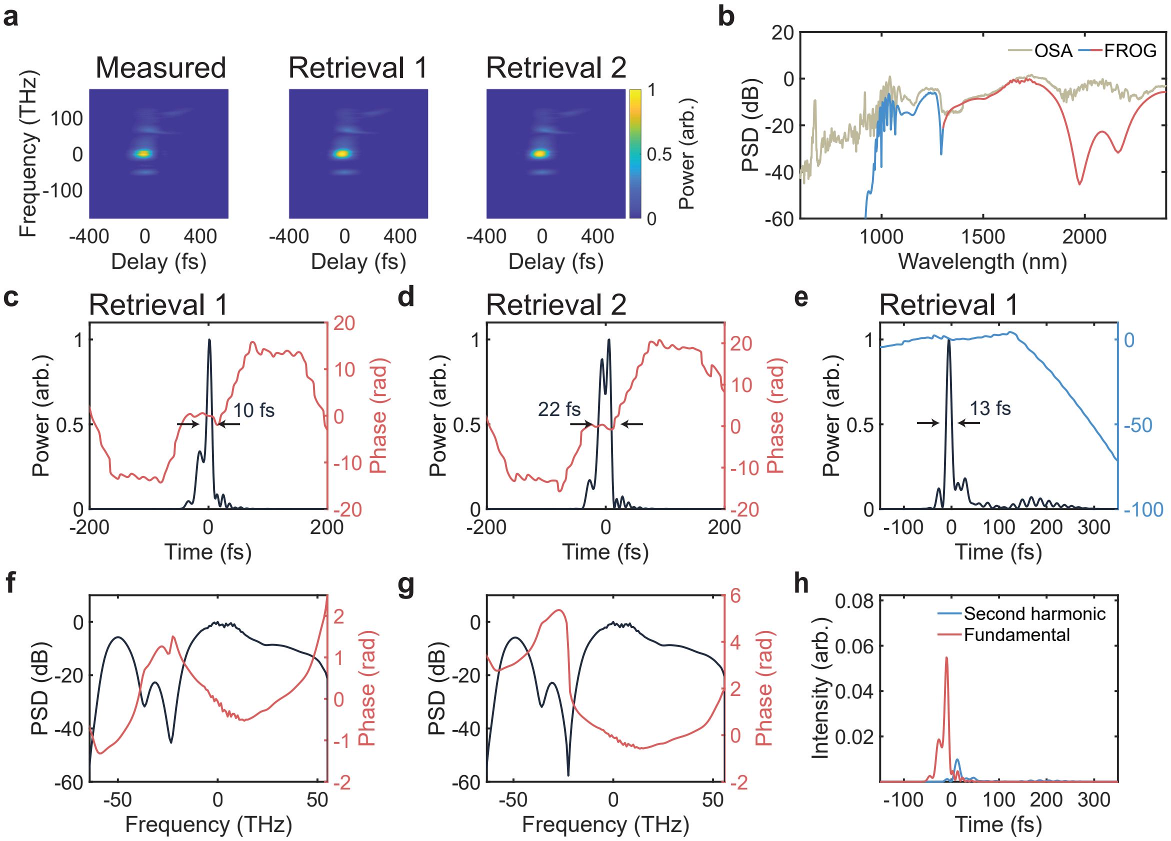

Figure S3: **Experimental FROG ambiguities.** **a**, Measured (left) SFG FROG spectrogram for an input energy of 5 pJ along with two example retrievals (right). **b**, Overlaid OSA and FROG spectra, showing good agreement. **c**, Fundamental pulse corresponding to retrieval 1. **d**, Fundamental pulse corresponding to retrieval 2. **e**, Second-harmonic pulse corresponding to retrieval 1. **f**, Fundamental spectrum corresponding to retrieval 1 and **g**, retrieval 2. **h**, Overlay of directly retrieved fundamental and second-harmonic temporal profiles. Both FROG retrievals show an error of 0.0044.

sharp feature is observed, whereas the double-humped pulse of retrieval 2 is a consequence of the two spectral bands being out of phase. A secondary issue is depicted in Fig. S3h. Although the fundamental and second-harmonic waves are expected to co-propagate, retrievals consistently indicate an approximately 20-fs separation between the two harmonics. We associate this observed separation with dispersion in the measurement path, which can be resolved by limiting the number of mirror bounces and propagation distance between the chip output and FROG.

We believe the observed phase ambiguity in the fundamental traces at high powers to be predominantly a consequence of the carrier-envelope offset frequency, f_{ceo}, of the driving laser being unlocked and non-zero. Because of this, the relative phase of the fundamental and and second-harmonic waveforms drifts from pulse to pulse, resulting in a non-repeating pulse train at the FROG. Such non-repeating pulse trains are known to cause ambiguities in FROG and have been the subject many experimental and numerical investigations[8,9]. To illustrate the consequence of the unlocked f_{ceo} on our FROG retrieval, we numerically construct a spectrogram which approximates the simulated spectrogram. This is by averaging the spectrograms generated from five distinct simulated waveforms made by summing the simulated fundamental and second-harmonic components with different relative phases. In our simulation, we assume an input energy of 3.7 pJ. As in the synthesis proposal of Fig. 5 in the main text, the relative phase is varied by changing the input envelope phase, ϕ_{ω}. The simulated fundamental and second-harmonic pulses are shown in Figs. S4a and S4b, respectively.

The combined spectral amplitudes and phases for the five simulated values of the input phase are shown in Figs. S4c and S4d. As may be expected, the spectral amplitudes and phases are similar everywhere except for in the overlap region around 230 THz. Here, interference results in significant spectral amplitude variation, and the spectral phases are observed to change at different rates. The average spectrogram constructed from these different waveforms is shown in Fig. S4e along with three different example retrieved spectrograms. The retrieval algorithm

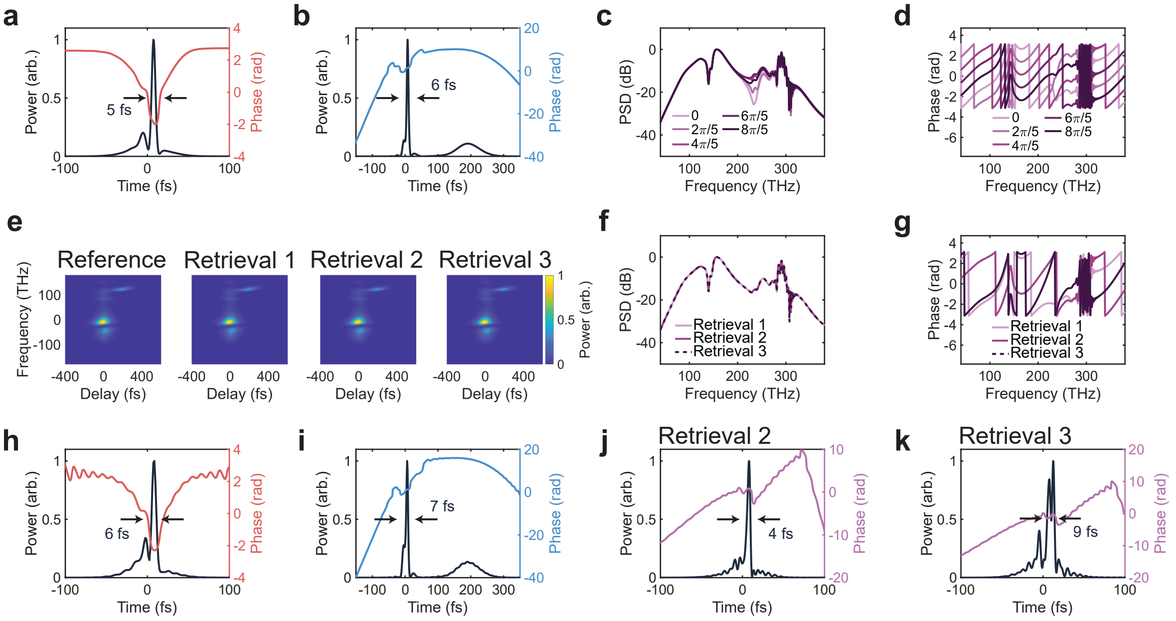

Figure S4: **Simulation of FROG ambiguity.** **a**, Simulated fundamental and **b**, second-harmonic waveforms for 3.7 pJ of pump pulse energy. **c**, Combined spectral amplitudes for different fundamental envelope phases. **d**, Corresponding spectral phases. **e**, Numerically constructed ambiguous FROG trace along with three example retrievals. **f**, Retrieved spectral amplitudes and **g**, phases 2. **h**, Retrieved fundamental and **i**, second harmonic pulses. **j**, Combined pulses for retrieval 2 and **k**, retrieval 3. FROG retrievals 1, 2, and 3 show errors of 0.00037, 0.00046, and 0.00067, respectively.

is run for 1000 iterations, resulting in similar errors for the retrieved spectrograms of 0.00037, 0.00046, and 0.00067 for retrievals 1, 2, and 3, respectively. As shown in Figs. S4f and S4g, all three retrievals result in similar spectral amplitudes; however, significant spectral phase variation in the vicinity of 230 THz is observed across retrievals.

The consequences of this are illustrated in Figs. S4h-k. In all cases, the fundamental and second harmonic temporal profiles are pretty faithfully reconstructed; examples corresponding to retrieval 1 are shown in Figs. S4h and S4i. However, vastly different combined temporal profiles may be observed, corresponding to the different relative phases between the fundamental and second harmonic components, as demonstrated by the examples of Figs. S4j and S4k. This ambiguity mirrors that of the experimentally measured fundamental pulses; however, it is not entirely clear why the ambiguity occurs experimentally in the middle of the fundamental spectrum rather than between the two harmonics. We believe that it is due to there being low SNR in this region due to the significant dip in spectral amplitude. This results in the optimal retrieval misattributing the phase ambiguity to this central dip in the fundamental spectrum. Such an artifact is consistent with phase ambiguities that typically exist between spectrally separated components in FROG measurements[10].

1.4 Device Design and Characterization

We design our device according to the design principles laid out in Section 2, and also in the theory section of the manuscript, and refer the reader to those sections for definitions of the parameters. We wish to operate in the $\alpha \approx 1$ regime with small Δk, such that similar soliton pulse widths and amplitudes may be observed at both the fundamental and second harmonics. This requires a low group velocity mismatch between the two harmonics to ensure small δ. In addition, we wish for the group velocity dispersion (GVD) at 2 μm to be sufficiently small so that the characteristic timescale of the system is on the order femtoseconds at moderate

input pulse energies in the pJ range. This demonstrates the feasibility of performing soliton compression towards the single-cycle regime using available integrated pulsed laser sources[11].

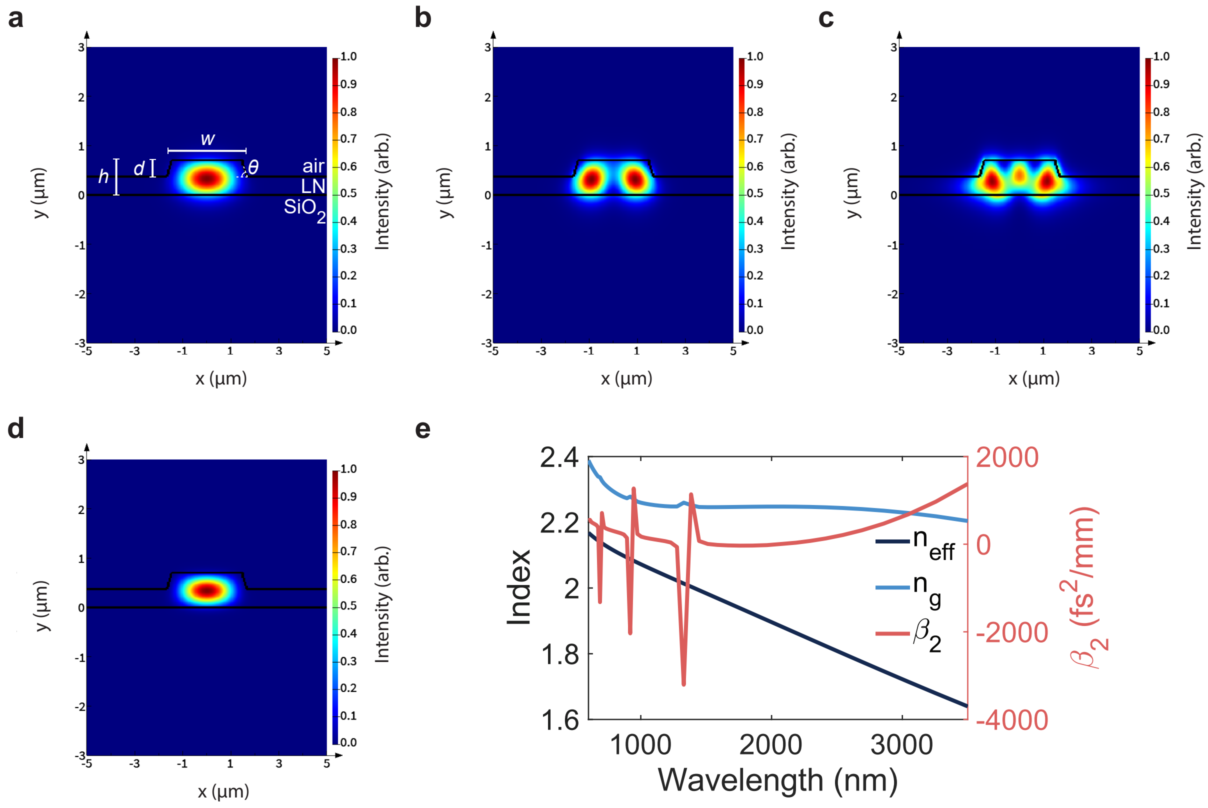

Figure S5: **Waveguide design.** **a**, TE0 mode at 2090 nm, with key design parameters indicated. **b**, TE1 mode at 2090 nm. **c**, TE2 mode at 2090 nm. **d**, TE0 mode at 1045 nm. **e**, Dispersion parameters for TE0 mode.

The key parameters contributing to the dispersion of our device are shown in Fig. S5a. They include the thin-film thickness, h, etch depth, d, and waveguide top width, w. Our fabrication process additionally results in a sidewall angle, θ, of 60°, which must also be accounted for. Using Lumerical, we sweep these three main parameters to find a geometry that satisfies our design requirements. The fabricated device geometry has h = 709 nm, d = 330 nm, and w = 2938 nm. With these parameters, the waveguide supports 3 TE modes at the fundamental wavelength of 2090 nm, the electric field intensities of which are shown in Figs. S5a, S5b, and

S5c. The fundamental mode at the second-harmonic wavelength of 1045 nm is shown in Fig. S5d.

We also use Lumerical to calculate the effective index, n_{eff}, group index, n_{g}, and GVD, β_2, of the fundamental mode as a function of wavelength. The results are shown in Fig. S5e. From this, we find the GVD at 2090 nm to be 9 fs^2 mm^{-1}, and at 1045 nm to be 141 fs^2 mm^{-1}. The group velocity mismatch (GVM) between the two is calculated to be 27 fs mm^{-1}. Spikes in the dispersion around 1350 nm, 930 nm, and 690 nm are the result of mode crossings near these wavelengths. However, all occur sufficiently far away from the fundamental and second harmonic carriers as to not significantly impede the broadening. Additionally, the poling period required for phase-matched SHG is calculated to be 5.69 μm from the effective indices of the fundamental and second harmonic, 1.88034 and 2.06409 respectively. The measured phase-mismatched device is designed to have a poling length of 6.5 mm with a period of 5.73 μm.

As mentioned in the main text, some of our input power is coupled into the higher-order modes at 2090 nm, resulting in there being content at 2090 nm in the OSA measurement that is not observed in the FROG retrieval. This is because the higher-order modes are temporally gated out in the X-FROG geometry. To illustrate this, we calculate the expected temporal separation between the TE0 and TE1 modes at the chip output. From the group indices of the two modes, we calculate a group velocity mismatch of 258 fs mm^{-1}. This results in there being a temporal separation of about 2 ps over the 8-mm-long device. This is well-separated from the X-FROG trace, which spans about 1 ps of time delay, centered about the TE0 mode. As the higher-order modes are not phase-matched for efficient nonlinear conversion, their spectra do not change significantly from that of the input, accounting for the additional spectral content around 2090 nm in the OSA trace.

We fabricate the device following the procedure described in ref. [12]. It is fabricated on X-cut MgO-doped thin-film lithium niobate on a SiO_2/Si substrate (NANOLN). To achieve the

periodic poling, we pattern Cr poling electrodes using lift-off. By applying a voltage across the electrodes, we periodically flip the ferroelectric domains. Following poling, we etch the waveguides using Ar-milling with hydrogen silsesquioxane (HSQ) as the etch mask. Finally, we mechanically polish the waveguide facets to enable end-fire coupling into the devices.

After fabrication, we characterize the throughput of our device and from them estimate the coupling losses. From free space to free space, we observe a 22-dB loss at 2090 nm through the chip. Based on the index contrast at the output, we expect a 10% reflection at the output facet of the chip. Furthermore, based on the NA of our output objective (Thorlabs LMM40X-P01), which is 0.5, we use Lumerical to estimate the power coupling between the fundamental TE mode and a Gaussian beam which fills the objective and is focused to the waveguide output facet. A more accurate calculation may be achieved by using a slightly elliptical free-space mode, but we believe the symmetric Gaussian mode to be a fair choice given that the $1/e^2$ width of the fundamental TE mode is close to the diffraction-limited beam waist, which we calculate as 1.33 µm. With these assumptions, the power coupling is 0.43. Finally, we must include the obscuration factor, which is reported to be 24% for the objective. Approximating the chip output as an ideal spherical wavefront, this obscuration factor can be directly taken as the loss. Based on these values, our output coupling is calculated to be 5.3 dB, which is similar to the output coupling that has previously been measured from our devices using other techniques. In our analysis, we round this value up to 6 dB, accounting for other non-idealities in our system such as scattering loss due to imperfect polishing, and use 16 dB as the input coupling calibration factor between off-chip and on-chip powers.

2 Theoretical Analysis

2.1 Soliton Solutions to Coupled Wave Equations

We begin our analysis with the coupled wave equations[13,14], written as:

$$\frac{\partial A_\omega}{\partial z} = i\kappa A_{2\omega} A_\omega^* e^{-i\Delta k z} - \frac{i\beta_\omega^{(2)}}{2}\frac{\partial^2 A_\omega}{\partial t^2}, \tag{S2a}$$

$$\frac{\partial A_{2\omega}}{\partial z} = i\kappa A_\omega^2 e^{i\Delta k z} - \Delta\beta' \frac{\partial A_{2\omega}}{\partial t} - \frac{i\beta_{2\omega}^{(2)}}{2}\frac{\partial^2 A_{2\omega}}{\partial t^2}, \tag{S2b}$$

where $A_\omega(z,t)$ and $A_{2\omega}(z,t)$ represent the amplitudes of the fundamental and second harmonic waves at frequencies ω and 2ω, respectively, normalized such that the instantaneous power in each wave is given by $|A_j|^2, j\epsilon\{\omega, 2\omega\}$. The time coordinate is defined such that the reference frame is co-moving at the group velocity of the fundamental wave. The phase mismatch parameter is $\Delta k = \frac{2\pi}{\Lambda} + 2k_\omega - k_{2\omega}$, where Λ is the poling period. $\kappa = \frac{\sqrt{2\eta_0}\omega d_{\text{eff}}}{n_\omega \sqrt{A_{\text{eff}}} n_{2\omega} c}$ is the nonlinear coupling coefficient, where d_{eff} is the effective nonlinearity, n_j is the refractive index of wave j, A_{eff} is the effective mode area, c is the speed of light, and η_0 is the impedance of free space. The group velocity mismatch is given by $\Delta\beta' = \frac{1}{v_{\text{g},2\omega}} - \frac{1}{v_{\text{g},\omega}}$, where $v_{\text{g},j}$ is the group velocity of wave j. Finally, $\beta_j^{(2)}$ is the group velocity dispersion of the jth wave. For the purposes of this analysis, we neglect higher dispersion orders.

We next introduce the normalized waves, defined as $a_\omega = -\frac{\sqrt{2}\kappa}{\beta}\sqrt{\left|\frac{\beta_\omega^{(2)}}{\beta_{2\omega}^{(2)}}\right|} A_\omega e^{-i\beta z}$ and $a_{2\omega} = \frac{\kappa}{\beta} A_{2\omega} e^{-i(2\beta+\Delta k)z}$, where β accounts for shifts in the phase velocity induced by the nonlinear interaction[15]. Additionally, we define a new spatial coordinate $\zeta = |\beta| z$ and a new temporal coordinate $\xi = \sqrt{\left|\frac{2\beta}{\beta_\omega^{(2)}}\right|}t$. Note that we have implicitly required that β be real. Although our analysis focuses on two-color bright soliton solutions, we note also that a wide variety soliton solutions exists in other parameter regimes. With these qualifications, we proceed with making the substitutions, yielding the following system of equations,

$$-is_1 \frac{\partial a_\omega}{\partial \zeta} = s_2 \frac{\partial^2 a_\omega}{\partial \xi^2} - a_\omega + a_{2\omega} a_\omega^*, \tag{S3a}$$

$$-is_1 \frac{\partial a_{2\omega}}{\partial \zeta} = s_3 \left|\frac{\beta_{2\omega}^{(2)}}{\beta_\omega^{(2)}}\right| \frac{\partial^2 a_{2\omega}}{\partial \xi^2} + is_3 \Delta\beta' \sqrt{-\frac{2}{\beta_\omega^{(2)}\beta}} \frac{\partial a_{2\omega}}{\partial \xi} - (2 + \frac{\Delta k}{\beta}) a_{2\omega} + \left|\frac{\beta_{2\omega}^{(2)}}{\beta_\omega^{(2)}}\right| \frac{a_\omega^2}{2}. \tag{S3b}$$

Here, $s_1 = \text{sgn}(\beta)$, $s_2 = -\text{sgn}(\frac{\beta_\omega^{(2)}}{\beta})$, and $s_3 = -\text{sgn}(\frac{\beta_{2\omega}^{(2)}}{\beta})$. In what follows, we take s_2 = s_3 = 1, requiring that $\beta_\omega^{(2)}$ and $\beta_{2\omega}^{(2)}$ share the same sign, opposite that of β, as this is known to yield bright soliton solutions[16]. Finally, defining $\sigma = \left|\frac{\beta_\omega^{(2)}}{\beta_{2\omega}^{(2)}}\right|$, $\delta = s_1 \Delta\beta' \sqrt{\left|\frac{2\beta_\omega^{(2)}}{(\beta_{2\omega}^{(2)})^2 \beta}\right|}$, and $\alpha = \sigma(2 + \frac{\Delta k}{\beta})$, we arrive at the normalized coupled wave equations,

$$-is_1 \frac{\partial a_\omega}{\partial \zeta} = \frac{\partial^2 a_\omega}{\partial \xi^2} - a_\omega + a_{2\omega} a_\omega^*, \tag{S4a}$$

$$-is_1 \sigma \frac{\partial a_{2\omega}}{\partial \zeta} = \frac{\partial^2 a_{2\omega}}{\partial \xi^2} + i\delta \frac{\partial a_{2\omega}}{\partial \xi} - \alpha a_{2\omega} + \frac{a_\omega^2}{2}. \tag{S4b}$$

Let us first study the soliton solutions with $\delta = 0$, assuming no walk-off between the fundamental and second-harmonic waves. Setting the spatial derivatives on the left-hand side of Eqs. S4a and S4b to 0, we have

$$0 = \frac{\partial^2 a_\omega}{\partial \xi^2} - a_\omega + a_{2\omega} a_\omega^*, \tag{S5a}$$

$$0 = \frac{\partial^2 a_{2\omega}}{\partial \xi^2} - \alpha a_{2\omega} + \frac{a_\omega^2}{2}. \tag{S5b}$$

To begin, we find the continuous-wave solutions, $a_{j,CW}, j\epsilon\{\omega, 2\omega\}$, by setting the time derivatives to 0. Assuming real solutions, which requires $\alpha > 0$, we find the following non-zero solutions,

$$a_{2\omega,CW} = 1, \tag{S6a}$$

$$a_{\omega,CW} = \pm\sqrt{2\alpha}. \tag{S6b}$$

The existence of two CW solutions with opposite phases at the fundamental wave is a well-known feature of degenerate quadratic nonlinear systems. For the analysis that follows, we will take the positive solution; however, the analysis would follow equivalently from the negative

solution. Secondarily, we see that the two waves have an amplitude ratio which is given by $\sqrt{2\alpha}$, the normalized phase mismatch parameter. Next, we seek particular solutions of the form

$$a_\omega(\xi) = a_{\omega,CW}\tilde{a}_\omega(\xi) = \sqrt{2\alpha}\tilde{a}_\omega(\xi), \tag{S7a}$$

$$a_{2\omega}(\xi) = a_{2\omega,CW}\tilde{a}_{2\omega}(\xi) = \tilde{a}_{2\omega}(\xi). \tag{S7b}$$

To proceed, we assume a large phase mismatch, $\alpha >> 1$, and perform an asymptotic expansion of $\tilde{a}_{2\omega}(\xi)$ and $\tilde{a}_\omega(\xi)$ in orders of the small parameter $\frac{1}{\alpha}$, yielding

$$\tilde{a}_j(\xi) = \tilde{a}_{j,0}(\xi) + \frac{1}{\alpha}\tilde{a}_{j,2}(\xi) + \frac{1}{\alpha^2}\tilde{a}_{j,3}(\xi) + ... \tag{S8}$$

Using this expansion in conjunction with Eqs. S7a and S7b and plugging into Eqs. S5a and S5b gives the following system of equations for terms of order $(\frac{1}{\alpha})^0$,

$$0 = \frac{\partial^2 \tilde{a}_{\omega,0}}{\partial \xi^2} - \tilde{a}_{\omega,0} + \tilde{a}_{2\omega,0}\tilde{a}^*_{\omega,0}, \tag{S9a}$$

$$0 = -\tilde{a}_{2\omega,0} + \tilde{a}^2_{\omega,0}. \tag{S9b}$$

Plugging Eq. S9b into S9a gives a second-order differential equation for $\tilde{a}_{\omega,0}$,

$$0 = \frac{\partial^2 \tilde{a}_{\omega,0}}{\partial \xi^2} - \tilde{a}_{\omega,0} + \tilde{a}_{\omega,0}|\tilde{a}_{\omega,0}|^2, \tag{S10}$$

which closely resembles the nonlinear Schrödinger equation governing cubic nonlinear optical systems[17]. Solving for $\tilde{a}_{\omega,0}$ assuming $\tilde{a}_{\omega,0}$ real gives the bright soliton solution,

$$\tilde{a}_{\omega,0}(\xi) = \sqrt{2}\,\mathrm{sech}(\xi), \tag{S11a}$$

$$\tilde{a}_{2\omega,0}(\xi) = 2\,\mathrm{sech}^2(\xi). \tag{S11b}$$

From this, we see that the temporal duration of the soliton solution is given by the characteristic timescale of the system, $\left|\frac{2\beta}{\beta_\omega^{(2)}}\right|$. This first-order solution of the asymptotic expansion is what has been predominantly studied in works on quadratic soliton pulse compression, due to its similarity to the fundamental Kerr soliton. Next, we may repeat the procedure to find solutions for higher orders of $\frac{1}{\alpha}$. For brevity, we directly write here the solutions for $(\frac{1}{\alpha})^1$ and $(\frac{1}{\alpha})^2$. For $(\frac{1}{\alpha})^1$, we have

$$\tilde{a}_{\omega,1}(\xi) = 2\sqrt{2}\,\mathrm{sech}(\xi)\tanh^2(\xi), \tag{S12a}$$

$$\tilde{a}_{2\omega,1}(\xi) = 4\,\mathrm{sech}^2(\xi)[4 - 5\,\mathrm{sech}^2(\xi)], \tag{S12b}$$

while for $(\frac{1}{\alpha})^2$, we see

$$\tilde{a}_{\omega,2}(\xi) = \frac{2\sqrt{2}}{3}\mathrm{sech}(\xi)[28\,\mathrm{sech}^4(\xi) + 32\,\mathrm{sech}^2(\xi) - 97], \tag{S13a}$$

$$\tilde{a}_{2\omega,2}(\xi) = \frac{8\sqrt{2}}{3}\mathrm{sech}^2(\xi)[181\,\mathrm{sech}^4(\xi) - 106\,\mathrm{sech}^2(\xi) - 76]. \tag{S13b}$$

Together, then, the asymptotic expansion in $\frac{1}{\alpha}$ for the soliton solutions of the coupled wave equations is given by

$$a_\omega(\xi) = 2\sqrt{\alpha}\,\mathrm{sech}(\xi)\Bigg(1 + \frac{2}{\alpha}\tanh^2(\xi) + \frac{2}{3\alpha^2}[28\,\mathrm{sech}^4(\xi) + 32\,\mathrm{sech}^2(\xi) - 97] + O(\frac{1}{\alpha^3})\Bigg), \tag{S14a}$$

$$a_{2\omega}(\xi) = 2\,\mathrm{sech}^2(\xi)\Bigg(1 + \frac{2}{\alpha}[4 - 5\,\mathrm{sech}^2(\xi)] + \frac{4\sqrt{2}}{3\alpha^2}[181\,\mathrm{sech}^4(\xi) - 106\,\mathrm{sech}^2(\xi) - 76] + O(\frac{1}{\alpha^3})\Bigg). \tag{S14b}$$

While this asymptotic solution works well to first order for cases when $\alpha >> 1$ and can be made more exact for cases with $\alpha \approx 1$ by including higher-order terms, it would be preferable to have a more succinct expression for the soliton solution for arbitrary α. Such a solution may be obtained by making the observation that the soliton solutions in both the pump and signal take the form of powers of sech functions, and that they share the same approximate pulse width. Thus, we make make the following ansatz[18],

$$a_{\omega}(\xi) = a_{\omega,0}\,\mathrm{sech}^{p}(\frac{\xi}{\tau}), \tag{S15a}$$

$$a_{2\omega}(\xi) = a_{2\omega,0}\,\mathrm{sech}^{q}(\frac{\xi}{\tau}), \tag{S15b}$$

where $a_{\omega,0}$ and $a_{2\omega,0}$ are the pulse amplitudes, assumed to be real, and τ will define the pulse width scaling. Plugging into the system of Eqs. S5a and S5b gives

$$\frac{a_{\omega,0}}{\tau^2}[p^2\,\mathrm{sech}^{p}(\frac{\xi}{\tau}) - (p^2+p)\,\mathrm{sech}^{(p+2)}(\frac{\xi}{\tau})] - a_{\omega,0}\,\mathrm{sech}^{p}(\frac{\xi}{\tau}) + a_{\omega,0}a_{2\omega,0}\,\mathrm{sech}^{p+q}(\frac{\xi}{\tau}) = 0, \tag{S16a}$$

$$\frac{a_{2\omega,0}}{\tau^2}[q^2\,\mathrm{sech}^{q}(\frac{\xi}{\tau}) - (q^2+q)\,\mathrm{sech}^{(q+2)}(\frac{\xi}{\tau})] - \alpha a_{2\omega,0}\,\mathrm{sech}^{q}(\frac{\xi}{\tau}) + \frac{a_{\omega,0}^2}{2}\,\mathrm{sech}^{2p}(\frac{\xi}{\tau}) = 0. \tag{S16b}$$

From here, we note that the first equation can be solved exactly when q = 2, requiring that $\tau = p$, and the second-harmonic pulse amplitude then follows as

$$a_{2\omega,0} = 1 + \frac{1}{p}. \tag{S17}$$

With these constraints, one may find an exact solution[19] using Eq. S16b when p = 2, corresponding to $\alpha = 1$. In this case, the solution is given by

$$a_\omega(\xi) = \frac{3}{\sqrt{2}}\,\mathrm{sech}^2(\frac{\xi}{2}), \tag{S18a}$$

$$a_{2\omega}(\xi) = \frac{3}{2}\,\mathrm{sech}^2(\frac{\xi}{2}). \tag{S18b}$$

However, an approximate analytical solution which gives exactly the behavior around the pulse peak and tails may be derived by making a few observations. The first is that, at the pulse peak ($\xi = 0$), all of the sech terms go to 1. Thus, satisfaction of Eq. S16b at $\xi = 0$ requires that

$$a_{\omega,0}^2 = 2a_{2\omega,0}(\frac{2}{p^2} + \alpha). \tag{S19}$$

Here, we note also that Eq. S16b is already satisfied at the tails of the pulses, as all of the sech terms approach 0 as $\xi \to \pm\infty$. The second observation that can be made is that the normalized Eqs. S5a and S5b exactly describe a particle in a potential U, where

$$U = \frac{a_\omega^2 a_{2\omega}}{2} - \frac{1}{2}a_\omega^2 - \frac{1}{2}\alpha a_{2\omega}^2. \tag{S20}$$

The Hamiltonian for the system is

$$H = \frac{1}{2}\left(\frac{\partial a_\omega}{\partial \xi}\right)^2 + \frac{1}{2}\left(\frac{\partial a_{2\omega}}{\partial \xi}\right)^2 + U. \tag{S21}$$

For such a conservative system, we expect the Hamiltonian to remain constant for all values of ξ. By noting that at the wings, where $\xi \to \pm\infty$, $H \to 0$, we see that this requires $H = 0$ for all values of ξ. As such, correct behavior at the pulse peak, where $\frac{\partial a_\omega}{\partial \xi} = \frac{\partial a_{2\omega}}{\partial \xi} = 0$, requires $H|_{\xi=0} = U|_{\xi=0} = 0$. This leads to a second equation,

$$a_{\omega,0}^2 a_{2\omega} - a_{\omega}^2 - \alpha a_{2\omega}^2 = 0. \tag{S22}$$

We may combine the above arguments along with Eqs. S17, S19, and S22 to arrive at the following set of soliton solutions, originally proposed by Sukhorukov[18], and repeated in the main text as Eqs. 1-5,

$$a_{\omega}(\xi) = a_{\omega,0}\,\text{sech}^p(\frac{\xi}{p}), \tag{S23a}$$

$$a_{2\omega}(\xi) = a_{2\omega,0}\,\text{sech}^2(\frac{\xi}{p}), \tag{S23b}$$

where the scaling behaviors for the parameters $a_{2\omega,0}$, $a_{\omega,0}$, and p are given as

$$p = \frac{1}{a_{2\omega,0} - 1}, \tag{S24a}$$

$$a_{\omega,0}^2 = \frac{\alpha a_{2\omega,0}^2}{a_{2\omega,0} - 1}, \tag{S24b}$$

$$\alpha = \frac{4(a_{2\omega,0} - 1)^3}{2 - a_{2\omega,0}}. \tag{S24c}$$

Having found the soliton solutions analytically, we briefly comment on the terminology used in the manuscript. In much of the literature around quadratic solitons, the language of "effective Kerr" is typically used to describe the situation where $\alpha >> 1$, since the dynamics at the fundamental are well-approximated by the nonlinear Schrödinger equation describing Kerr systems. As large α has typically been coincident with large Δk, where rapid back-and-forth conversion is observed between the fundamental and second-harmonic waves, this regime is also sometimes referred to as the "cascading limit."

However, care must be taken with respect to this language in the context of the temporal solitons considered here. Firstly, since α additionally depends on σ, it is possible to operate in the effective Kerr regime in a system with large σ and small Δk through shaping the dispersion. Likewise, one may design for small σ such that α is small even for large Δk, in which case the shape of the fundamental soliton is not well-predicted by the effective Kerr framework despite the large phase mismatch. Thus, there is some ambiguity in using the terms "effective Kerr" and "cascading limit" interchangeably; to avoid this ambiguity, we therefore refer specifically to the case of large α as the "effective Kerr" regime or "effective Kerr limit" and separately distinguish between phase-matched and phase-mismatched operation. As emphasized in our manuscript, we wish both to operate away from the effective Kerr limit, with $\alpha \approx 1$, and with Δk small, such that the fundamental and second-harmonic pulses exhibit similar temporal profiles and significant energy transfer to the second harmonic is also achieved.

2.2 Numerical Soliton Solutions

We numerically find the soliton solutions using Newton's Method with the solutions given by Eqs. S23 and S24 as a seed. To do so, we encode the two equations for which we wish to find the zeros, Eqs. S5a and S5b, where we numerically compute derivatives in ξ in the Fourier domain using the Fast Fourier Transform. For a Fourier grid of size N, the state vector a_{curr} is size 2N, consisting of the value of a_ω at each of the N points in ξ along with the value of $a_{2\omega}$ at all N points. Newton's Method requires iteratively computing the 2N-by-2N Jacobian J, composed by taking partial derivatives of the Eqs. S5a and S5b with respect to the 2N points in the current state vector, and inverting it to compute $a_{\text{next}} = a_{\text{curr}} - J^{-1} f(a_{\text{curr}})$. Here, $f(a_{\text{curr}})$ is a vector of size 2N consisting of the solutions of Eqs. S5a and S5b for the 2N values of the state vector a_{curr}.

The numerically computed soliton solutions for different values of α along with scaling be-

haviors of the soliton pulse amplitude and full width at half maximum (FWHM) are shown in Fig. S6. The pulse profiles are shown in Figs. S6a and S6d for the fundamental and second harmonic, respectively. As noted in the main text, we see that at large values of α, the normalized fundamental amplitude greatly exceeds that of the second harmonic and vice versa for small values of α. This behavior is shown more clearly in Figs. S6b and S6e, where the pulse amplitudes are plotted as a function of α. The numerically computed solutions are shown by dark dashed lines, and exhibit near-perfect agreement with the light, solid lines showing the analytic solutions of Eqs. S23 and S24. Similarly, we plot the analytic and numerically computed soliton FWHM for both waves in Figs. S6c and S6f. Again, good agreement between the numeric and analytic solutions is observed, particularly for large α. Here, we see that the FWHM is relatively stable for $\alpha > 1$ as the asymptotic solution of Eqs. S14a and S14b is approached but quickly grows for $\alpha < 1$.

After solving for the system of Eqs. S5a and S5b, we additionally use numerical continuation to find solutions to the modified system where $\delta \neq 0$, given by setting the right-hand side of Eqs. S4a and S4b equal to 0, yielding

$$0 = \frac{\partial^2 a_\omega}{\partial \xi^2} - a_\omega + a_{2\omega} a_\omega^*, \tag{S25a}$$

$$0 = \frac{\partial^2 a_{2\omega}}{\partial \xi^2} + i\delta \frac{\partial a_{2\omega}}{\partial \xi} - \alpha a_{2\omega} + \frac{a_\omega^2}{2}. \tag{S25b}$$

Here, we begin with the solutions found using Newton's Method at $\delta = 0$ and find the solution for increasing values of δ, using the solution for the previous value of δ as a seed. An example of how the soliton solution changes for varying δ with fixed $\alpha = 1.64$ is given in Fig. S7. The real parts of the soliton solutions at the fundamental and second harmonic are shown in Fig. S7a and S7c, with corresponding imaginary parts shown in Fig. S7b and S7d. As may be observed, increasing δ results in a growing imaginary component of the soliton. In addition, the

real component is observed to skew; here, we've shown positive values of δ for which the skew shifts the center of mass in the direction of the positive time coordinate. However, we observe also that for sufficiently small values of δ, the soliton is still extremely well-approximated by the $\delta = 0$ solution.

At large values of δ, we note that our continuation algorithm no longer converges to a soliton solution but either blows up to infinity or converges to the trivial solution, $a_\omega = a_{2\omega} = 0$. To better understand where this transition occurs between the existence and non-existence of a stable soliton solution, we again consider the Eqs. S25a and S25b. A solution for $a_{2\omega}$ in terms of a_ω may be found through direct integration of Eq. S25b. Doing so yields the solution

$$a_{2\omega}(\xi) = \int_{-\infty}^{\infty} \frac{a_\omega^2(\xi - \xi')}{4\sqrt{\alpha - \delta^2/4}} e^{-i\frac{\delta}{2}} R_\pm(\xi') d\xi', \tag{S26}$$

where $R_+(\xi') = e^{-\sqrt{\alpha - \delta^2/4}|\xi'|}$ is used if $\alpha - \delta^2/4 \geq 0$ and $R_-(\xi') = \sin\left(\sqrt{\alpha - \delta^2/4}|\xi'|\right)$ is used if $\alpha - \delta^2/4 < 0$. Thus, we see that a non-oscillatory soliton solution is expected only if $\alpha \geq \delta^2/4$, which has previously been referred to as the stationary regime of pulse compression[20]. To confirm that this analysis is consistent with what we observe numerically, we plot (Fig. S8a) the soliton existence (dark blue) and non-existence regimes (light blue), as determined by the convergence of our Newton's Method to a non-zero stationary solution, as a function of α and δ. The analytically computed boundary, $\alpha = \delta^2/4$ is shown by the solid, black line, in good agreement with our numerical solver.

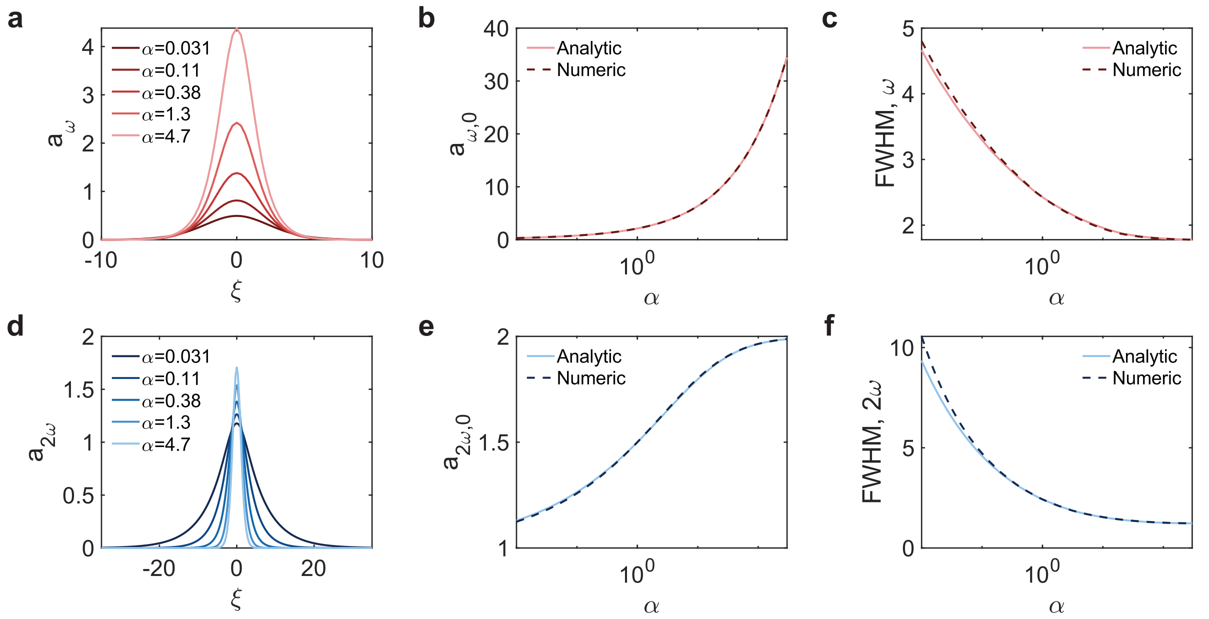

Figure S6: **Soliton solutions with** $\delta = 0$**. a**, Fundamental soliton solutions. **b**, Amplitude of fundamental soliton. **c**, FWHM of fundamental soliton. **d-f**, Corresponding plots for second-harmonic soliton. FWHM, full width at half maximum

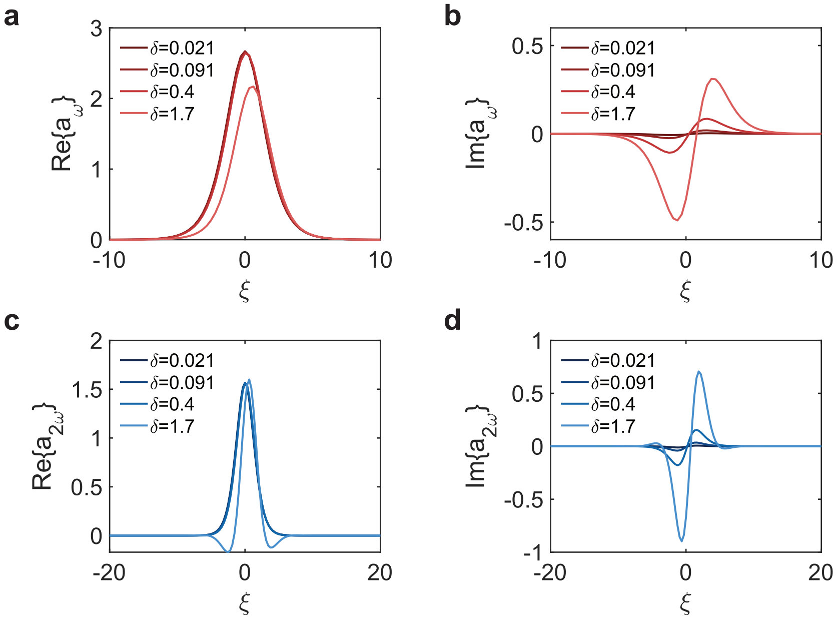

Figure S7: **Soliton solutions with** $\delta \neq 0$**. a**, Real and **b**, imaginary parts of fundamental soliton solutions. **c**, Corresponding real and **d**, imaginary parts of second-harmonic solitons.

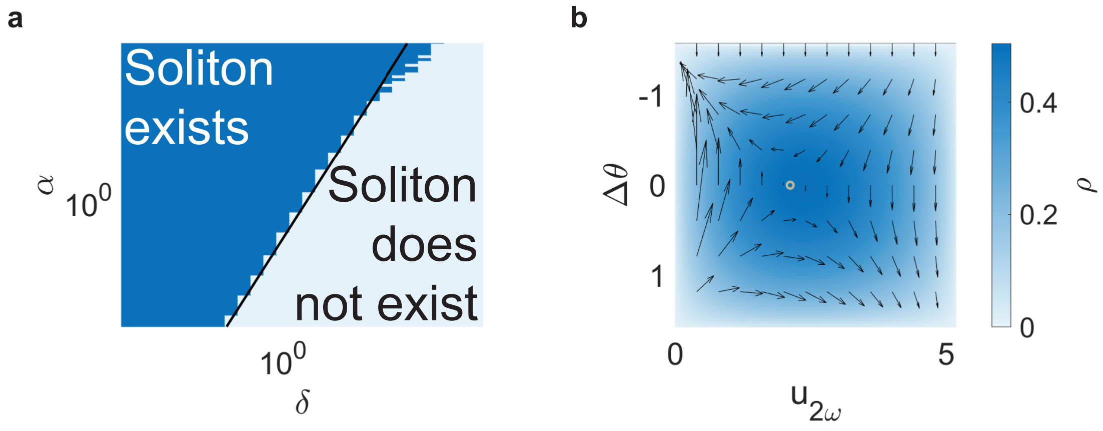

Figure S8: **Soliton existence and stability.** **a**, Soliton existence and non-existence regimes determined by convergence of Newton's method to a non-zero solution. The expected boundary $\alpha = \delta^2/4$ is given by the solid, black line. **b**, Phase space diagram with $\alpha = 1$ of the second harmonic soliton amplitude, $u_{2\omega}$, relative phase, $\Delta\theta$ found using the Lagrangian analysis. The color map shows the value of the pulse width parameter, ρ, corresponding to the given values of $u_{2\omega}$ and $\Delta\theta$. The soliton solution is indicated by the open tan circle.

2.3 Lagrangian Analysis

To better understand the stability of the soliton solution and the compression dynamics near the soliton solution, we perform a Lagrangian analysis[21] on the system of Eqs. S4a and S4b, setting $\delta = 0$. In this case, the Lagrangian density may be written as

$$L = \sigma\,\mathrm{Im}\left\{a_{2\omega}^* \frac{\partial a_{2\omega}}{\partial \zeta}\right\} + \mathrm{Im}\left\{a_\omega^* \frac{\partial a_\omega}{\partial \zeta}\right\} + \left|\frac{\partial a_\omega}{\partial \zeta}\right|^2 + \left|\frac{\partial a_{2\omega}}{\partial \zeta}\right|^2 + |a_\omega|^2 + \alpha |a_{2\omega}|^2 - \mathrm{Re}\left\{a_\omega^2 a_{2\omega}^*\right\}. \quad \text{(S27)}$$

To proceed, we assume the following simplified forms for the fundamental and second-harmonic waves based on the known exact soliton solution for $\alpha = 1$,

$$a_\omega(\zeta, \xi) = u_\omega(\zeta)\sqrt{\rho(\zeta)}\,\mathrm{sech}^2\big(\rho(\zeta)\xi\big)e^{i\frac{\theta_\omega(\zeta)}{2}}, \quad \text{(S28a)}$$

$$a_{2\omega}(\zeta,\xi) = u_{2\omega}(\zeta)\sqrt{\rho(\zeta)}\,\mathrm{sech}^2\big(\rho(\zeta)\xi\big)e^{i\theta_{2\omega}(\zeta)}, \tag{S28b}$$

where u_ω, $u_{2\omega}$, θ_ω, and $\theta_{2\omega}$ are the fundamental and second harmonic pulse amplitudes and phases, respectively, and ρ is the pulse width parameter, assumed to be the same for both the fundamental and second harmonic. The time-averaged Lagrangian density, $\mathcal{L}$, is then obtained inserting this ansatz into S27 and integrating over ξ:

$$\mathcal{L} = \int_{-\infty}^{\infty} L(\xi,\zeta)d\xi. \tag{S29}$$

Finally, the equations of motion for the system parameters can be found using the Euler-Lagrange equation, $\frac{\partial}{\partial\zeta}\big(\frac{\partial\mathcal{L}}{(\frac{\partial f}{\partial\zeta})}\big) = \frac{\partial\mathcal{L}}{\partial f}$, where $f \,\epsilon\, \{\rho,\theta_\omega,\theta_{2\omega},u_\omega,u_{2\omega}\}$. Steady-state solutions may be found by setting the resulting ζ derivatives to 0, yielding the following set of algebraic equations,

$$4\rho^{3/2}(u_{2\omega}^2 + u_\omega^2) = u_\omega^2 u_{2\omega}, \tag{S30a}$$

$$2 + \frac{8}{5}\rho^2 = \frac{8}{5}\sqrt{\rho}u_{2\omega}, \tag{S30b}$$

$$2\alpha + \frac{8}{5}\rho^2 = \frac{4}{5}\sqrt{\rho}\frac{u_\omega^2}{u_{2\omega}}. \tag{S30c}$$

In the case of $\alpha = 1$, the known soliton solution given by Eq. S18 is recovered exactly, as expected. Away from $\alpha = 1$, the system approximates the soliton solution but deviates slightly due to the assumption that the fundamental and second harmonic share the same pulse profile and width. The dynamics of the system can be reduced to two algebraic equations and two differential equations,

$$\rho^{3/2} = \frac{1}{4}\frac{u_{2\omega}u_\omega^2}{u_{2\omega}^2 + u_\omega^2}\cos(\Delta\theta), \tag{S31a}$$

$$\frac{u_\omega^2}{2} + \sigma u_{2\omega}^2 = \eta_{\text{sol}}, \tag{S31b}$$

$$\frac{du_{2\omega}}{d\zeta} = -\frac{2}{5\sigma}\sqrt{\rho}u_\omega^2 \sin(\Delta\theta), \tag{S31c}$$

$$\frac{d\Delta\theta}{d\zeta} = -\frac{2}{5}(\frac{u_\omega^2}{\sigma u_\omega} - 4u_{2\omega})\sqrt{\rho}\cos(\Delta\theta) + \frac{4}{5}(\frac{1}{\sigma} - 2)\rho^2 + (\frac{\alpha}{\sigma} - 2), \tag{S31d}$$

where we have re-parameterized the phase in terms of $\Delta\theta = \theta_\omega - \theta_{2\omega}$. Equation S31b is an energy conservation relation for the normalized system, with η_{sol} being a constant representing the total energy of the system.

The phase space diagram corresponding to Eqs. S31c and S31d for $\alpha = 1$ can be found in Fig. S8b. The color gradient in the background further shows the value of ρ, computed using the algebraic Eqs. S31a and S31b, where η_{sol} is computed from the soliton solution given by the system of Eqs. S30a-S30c. We observe that, in the space of the considered parameters, the soliton solution (open, tan circle) is a saddle point. We note also that the soliton solution corresponds very nearly to the largest value of ρ (corresponding to the minimum value of the pulse width). Although this simplified model does not capture the full compression behavior (for example, the observed back-and-forth conversion), the behavior is consistent with the overall compression dynamics of Fig. 1b. Specifically, we observe that the pulse nearly compresses to the soliton solution, around which pulse evolution is observed to be slow, but eventually begins to broaden again. This slow evolution, but ultimate instability, of the pulse near the soliton solution is consistent with the finding from the Lagrangian analysis that the soliton is a saddle point.

This analysis additionally highlights another important property of the soliton solution of the system, which is that $\Delta\theta = 0$ for the soliton state. We note here that in terms of the fundamental second harmonic envelope phases, respectively $\phi_\omega = \frac{\theta_\omega}{2}$ and $\phi_{2\omega} = \theta_{2\omega}$, this result implies that $2\phi_\omega = \phi_{2\omega}$ in the soliton state. Therefore, a change $\Delta\phi_\omega$ in the fundamental phase results in a change $\Delta\phi_{2\omega}$ that is twice as large. This is the basis of the synthesis method proposed in the

main text, as a simple modulation of the envelope phase of the fundamental input to the two-color compression can be used to directly shape the relative phase of the two output harmonics for the realization of a variety of waveforms.

2.4 Pulse Compression Simulations

To further explore the compression dynamics and provide finally some basic design guidelines, we perform Fourier split-step simulations of the coupled wave equations, Eqs. S4a and S4b, as described in the main text. To seed the simulations, we first take the numerically computed soliton solutions, as described in Section 2.2, and find the pulse width of the fundamental soliton as well as the total normalized energy in the combined fundamental and second harmonic soliton solutions. We then input a sech-shaped pulse at the fundamental with a pulse energy equal to the total soliton energy. To study the compression dynamics, we vary the ratio of the FWHM of the input, FWHM_{in}, to that of the soliton, FWHM_{sol} for different values of α, δ, and σ. For simplicity, we have assumed sgn$(\beta) = 1$ in our simulations, but identical scaling behaviors would be observed for sgn$(\beta) = -1$, with the primary difference being in the accumulated propagation phase.

The results of this analysis are used to generate the scaling behavior plots of Figs. 2c-f. Several supporting results are shown in Fig. S9. Figures S9a and S9b show the evolution of the fundamental and second harmonic as a function of the normalized propagation distance with $\alpha = 1.64$, $\sigma = \alpha/5$, and $\delta = 0$. This case represents compression in the more strongly phase-mismatched regime, where several back-and-forth conversions are observed before the waves approaches the soliton solution near the optimum compression point, ζ_{opt}, indicated by the dashed, white line. As mentioned in the main text, we define ζ_{opt} as the point at which the minimum pulse width is observed at the fundamental. Another interesting feature of note, as predicted by the Lagrangian analysis, is that the waves do not remain in the compressed

soliton state but are then observed to broaden. A second regime of compression is observed near $\zeta = 3\zeta_{\text{opt}}$, indicative of breathing dynamics for the compression system. By contrast, we show compression in the weakly phase-mismatched regime with $\sigma = \alpha/2.1$ in Figs. S9c and S9d. Here, only about two cycles of back-and-forth conversion are observed before ζ_{opt} is reached. However, the shape of the compressed pulse and optimum distance, ζ_{opt}, are observed to be similar in both the phase-mismatched and near phase-matched cases.

Having shown a few examples of the compression dynamics, we turn now to the claim that α is the dominant parameter in determining the compression behavior. In Fig. S9e, we show ζ_{opt} for many values of σ with constant $\alpha = 1.64$ and $\delta = 0$. Besides some small deviation for the largest value of σ, where the system is very nearly phase-matched, we observe extremely similar scaling behaviors, all of which follow well the fit of Eq. 6 in the main text. Likewise, we show the scaling behavior for different values of δ in Fig. S9f. Again, we see nearly identical scaling behavior besides the extreme case of large δ, where we are near the edge of the soliton existence regime.

Finally, as a probe into the stability of the compression system, we consider the quantity $\Delta\zeta_{\text{opt}}$, which we define as the length ζ for which the FWHM of the fundamental pulse remains within 5% of its value at ζ_{opt}. The ratio $\Delta\zeta_{\text{opt}}/\zeta_{\text{opt}}$ as a function of $\text{FWHM}_{\text{in}}/\text{FWHM}_{\text{sol}}$ is shown in Fig. S9g. Here, we see that the ratio $\Delta\zeta_{\text{opt}}/\zeta_{\text{opt}}$ scales inversely with the compression factor, indicating that the compression of longer pulses is more sensitive to noise at the input. This is consistent with intuitions from soliton pulse compression in Kerr systems. Furthermore, the behavior appears largely independent of α.

2.5 Theoretically-Informed Design Rules

Having now studied the soliton solutions of the normalized coupled wave equations, Eqs. S4a and S4b, and characterized the corresponding pulse compression behaviors, we may offer sev-

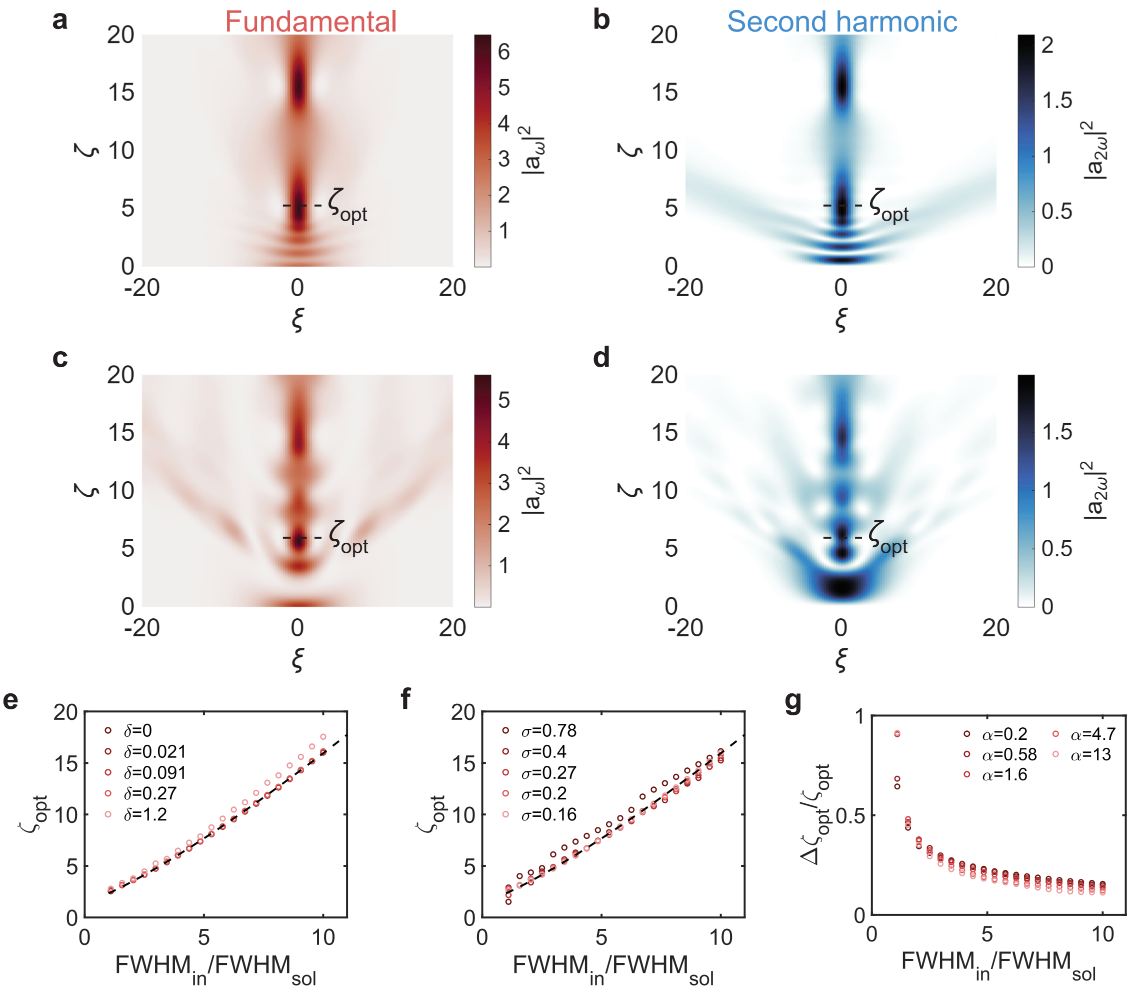

Figure S9: **Normalized compression results with constant $\alpha = 1.64$. a**, Phase-mismatched compression with $\sigma = \alpha/5$ for the fundamental and **b**, second harmonic. **c**, Nearly phase-matched compression with $\sigma = \alpha/2.1$ for the fundamental and **d**, second harmonic. The optimum compression length, ζ_{opt} is indicated in all four cases. **e**, ζ_{opt} as a function of $\mathrm{FWHM_{in}/FWHM_{sol}}$ for various values of σ, showing similar scaling behavior. **f**, Likewise, ζ_{opt} exhibits similar scaling behaviors for different values of δ. **g**, Ratio of $\Delta\zeta_{opt}$ to ζ_{opt} as a function of $\mathrm{FWHM_{in}/FWHM_{sol}}$.

eral design guidelines for achieving two-color soliton compression. There are several considerations for the design of the soliton compression system. The first is the desired pulse shape, which is fully determined by α, as discussed above. Secondly, one must consider the ratio of fundamental to second-harmonic peak power, which we recall is given in physical units by $\frac{|A_{\omega,0}|^2}{|A_{2\omega,0}|^2} = \left|\frac{\beta^{(2)}_{2\omega}}{2\beta^{(2)}_{\omega}}\right| \frac{|a_{\omega,0}|^2}{|a_{2\omega,0}|^2}$. Here, $A_{j,0}$ refers to the soliton amplitude in the jth wave, in direct analogy to the normalized soliton amplitudes, $a_{j,0}$. The third is the resultant pulse FWHM, which we have observed is on the order of the characteristic time of the system, $\sqrt{\left|\frac{\beta^{(2)}_{\omega}}{2\beta}\right|}$, for most reasonable values of α. Finally, we must satisfy the constraint $\alpha \geq \delta^2/4$, which translates in physical parameters to $\frac{2\left|\beta^{(2)}_{2\omega}\beta\right|}{(\Delta\beta')^2}(2 + \frac{\Delta k}{\beta}) \geq 1$.

Before continuing, we must also find an expression for β. One approach is to use conservation of energy. Specifically, we have that:

$$E_{\text{sol}} = \left|\frac{\beta}{\kappa}\right|^2 \left(\frac{1}{2\sigma}\int_{-\infty}^{\infty} a^2_{\omega}\left(\sqrt{\left|\frac{2\beta}{\beta^{(2)}_{\omega}}\right|}t\right)dt + \int_{-\infty}^{\infty} a^2_{2\omega}\left(\sqrt{\left|\frac{2\beta}{\beta^{(2)}_{\omega}}\right|}t\right)dt\right), \tag{S32}$$

where $a_{\omega}(\xi)$ and $a_{2\omega}(\xi)$, $\xi = \sqrt{\left|\frac{2\beta}{\beta^{(2)}_{\omega}}\right|}t$, may be taken as the soliton solutions of Eq. S23, and E_{sol} is, therefore, the total energy in the soliton solution. As we have observed, the majority of the pulse energy is retained in the soliton solution for moderate compression factors (less than a factor of ≈ 10). Thus, we may consider E_{sol} also to be the required input energy. This allows us therefore to relate β precisely to S32 for a given soliton solution.

With these key parameters in mind and a solution for β, we offer a suggested work flow for designing a compression system. The most stringent requirement is that the signs of $\beta^{(2)}_{\omega}$ and $\beta^{(2)}_{2\omega}$ must be the same to satisfy our initial assumptions for $s2$ and $s3$. After meeting this requirement, one should then try to minimize $\left|\Delta\beta'\right|$, as a smaller walk-off will give more flexibility on the other parameters of the system.

Once such a regime has been found, one may optimize for the desired soliton shape, FWHM,

and peak power ratio. As discussed above, the shape of the soliton solutions is determined by α. In the effective Kerr limit of large α, we observe a sech-shaped field envelope for the fundamental and a sech^2-shaped field envelope for the second harmonic. This results in narrower pulses for the second harmonic than the fundamental. Conversely, shorter pulses are observed at the fundamental in the case of small α. Both envelopes take the same sech^2 shape at $\alpha = 1$. In our experiment, we aim to operate near $\alpha = 1$ to achieve similar pulse widths at both harmonics.

After determining the desired value of α, one may consider the peak power ratio. By definition, we require $\sigma \leq \alpha/2$. If one selects $\beta_{\omega}^{(2)}$ and $\beta_{2\omega}^{(2)}$ such that $\sigma \approx \alpha/2$, very little phase-mismatch is required for achieving the desired compression, resulting in nearly equal distributions of power between the two pulses. This may be readily observed in the effective Kerr limit, where $\frac{|a_{\omega,0}|^2}{|a_{2\omega,0}|^2} = |\alpha| \approx |2\sigma| = \left|\frac{2\beta_{\omega}^{(2)}}{\beta_{2\omega}^{(2)}}\right|$. Then, in terms of physical parameters, we have that $\frac{|A_{\omega,0}|^2}{|A_{2\omega,0}|^2} = \left|\frac{\beta_{2\omega}^{(2)}}{2\beta_{\omega}^{(2)}}\right| \frac{|a_{\omega,0}|^2}{|a_{2\omega,0}|^2} = 1$. If one wishes, however, to compress the fundamental only with limited conversion to the second harmonic, then one should design for $\sigma << \alpha$, and a large Δk should be used. In our system, we have $\sigma = 0.065$, so we require a moderate Δk to operate near $\alpha = 1$.

While the desired peak power ratio informs the ratio of $\beta_{\omega}^{(2)}$ to $\beta_{2\omega}^{(2)}$, design of their absolute values is determined by the soliton existence condition, desired FWHM, and available pulse energy. Specifically, we see that a larger magnitude of $\beta_{2\omega}^{(2)}$ can compensate the walk-off $\Delta\beta'$ in satisfying the existence condition, $\frac{2\left|\beta_{2\omega}^{(2)}\beta\right|}{(\Delta\beta')^2}(2 + \frac{\Delta k}{\beta}) \geq 1$. However, with a fixed ratio of $\left|\frac{\beta_{\omega}^{(2)}}{\beta_{2\omega}^{(2)}}\right|$, this means also increasing $\left|\beta_{\omega}^{(2)}\right|$, which will result in a longer soliton pulse width for the same $|\beta|$. Offsetting this through increasing $|\beta|$ requires a larger input pulse energy. A well-optimized system will therefore maximize the magnitude of $\beta_{2\omega}^{(2)}$, while still maintaining that the desired pulse width is achievable in the range of available pump energies. This may be done iteratively through the use of Eq. S32. In our system, we have prioritized operation with a small $\left|\beta_{\omega}^{(2)}\right|$ to show that few-cycle pulse compression is attainable with pJ pump pulse energies, as may be

achieved with available integrated pulsed sources.

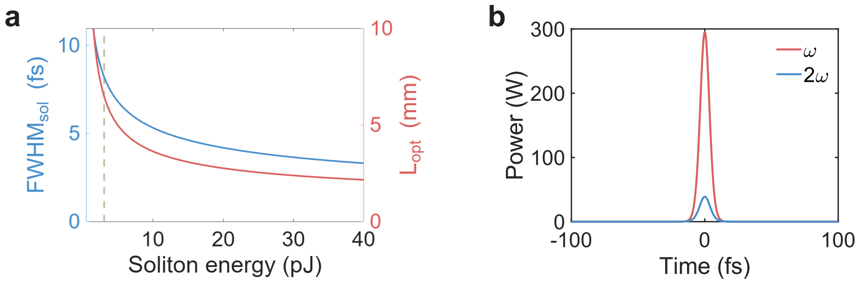

Figure S10: **Device design and soliton solution**. **a**, Predicted soliton FWHM and optimum device length as a function of input energy. The tan, dashed line shows the experimentally desired operation point. **b**, Predicted soliton solution corresponding to the experimental parameters.

Finally, having determined the other system parameters, one may calculate the required waveguide length. To do so, should take desired compression factor as the ratio of the input pulse FWHM to that of the desired soliton solution and use Eq. 6 of the main text to calculate the corresponding ζ_{opt}. The device length may then be directly calculated as $L_{\text{opt}} = \frac{\zeta_{\text{opt}}}{|\beta|}$. To illustrate this process for our own device parameters, we plot FWHM$_{\text{sol}}$ and L_{opt} as a function of the input energy in Fig. S10a. Our desired operation point is shown by the tan, dashed line, corresponding to a FWHM$_{\text{sol}}$ of 8 fs and L_{opt} of 6.5 mm. The resulting soliton solution is shown in Fig. S10b.

2.6 Relationship to Soliton Number

In cubic soliton compression systems, the many features of the compression are typically encapsulated by the soliton number, including the stability, compression factor, and compression quality. This formalism has also been shown to be accurate in an effective Kerr description of quadratic soliton compression systems, with large Δk, where an effective soliton number for the SHG process, N_{SHG}, may be readily obtained[22]. Here, we show how this effective soliton number arises from the soliton framework presented above and attempt to generalize it, which

may give a further analytical tool for understanding the dynamics of the quadratic soliton compression process. That such a generalization should be possible is indicated by the general insensitivity to α of the scaling behaviors for the two-color soliton compression process, as shown in Fig. 2 of the main text.

In Kerr systems, the soliton number, N, is defined by the ratio of the input pulse energy to the energy of the soliton solution with the same width as the pump (typically a sech-shaped pump is assumed)[23]. Soliton pulse compression occurs for $N > 1$. Likewise, we may define the effective soliton number as

$$N_{\text{SHG}} = E_{\text{in}}/E_{\text{sol}}. \tag{S33}$$

In general, defining $\tau_{\text{sol}} = \sqrt{\left|\frac{\beta_\omega^{(2)}}{2\beta}\right|}$, the energy in the soliton may be written from Eq. S32 in physical parameters as

$$E_{\text{sol}} = \frac{\left|\beta_\omega^{(2)}\right|^2}{2|\kappa|^2\tau_{\text{sol}}^4}\left(\frac{1}{2\sigma}\int_{-\infty}^{\infty} a_\omega^2\left(\frac{t}{\tau_{\text{sol}}}\right)dt + \int_{-\infty}^{\infty} a_{2\omega}^2\left(\frac{t}{\tau_{\text{sol}}}\right)dt\right). \tag{S34}$$

To find a simplified expression for the soliton number, we assume that the input shares the same temporal profile as the fundamental soliton. Then, we may write the energy of the input as

$$E_{\text{in}} = \frac{P_{\text{p,in}}}{a_{\omega,0}^2}\int_{-\infty}^{\infty} a_\omega^2\left(\frac{t}{\tau_{\text{sol}}}\right)dt, \tag{S35}$$

where the factor of $a_{\omega,0}^2$, given by S24, ensures a normalized temporal profile. Still, plugging into Eqn. S33 would yield a rather complicated expression. The situation is rectified if we assume operation with large Δk, where the contribution of the second harmonic to the overall output energy is negligible. Then, we have that

$$N_{\text{SHG}} \approx \frac{4\sigma|\kappa|^2\tau_{sol}^4}{\left|\beta_\omega^{(2)}\right|^2 a_{\omega,0}^2} P_{\text{p,in}}. \tag{S36}$$

In the effective Kerr limit of large α, where $a_{\omega,0}^2 \approx 4\alpha$, and assuming that $\frac{|\Delta k|\tau_{\text{sol}}^2}{\left|\beta_\omega^{(2)}\right|} >> 1$, we may further find that

$$N_{\text{SHG}} \approx \frac{|\kappa|^2\tau_{sol}^2}{2\left|\beta_\omega^{(2)}\right||\Delta k|} P_{\text{p,in}}, \tag{S37}$$

which is equivalent to the expression in ref. [22].

2.7 Full Simulation and Mapping to Experiment

For comparison with experiment, as explained in the Methods section of the main text, we directly simulate Eqs. S2a and S2b, but with the additional inclusion of loss terms for the fundamental and second harmonic, α_ω and $\alpha_{2\omega}$. As in the normalized simulations of Section 2.4, we employ a Fourier split-step simulation using a fourth-order Runge-Kutta method for the nonlinear step. A full list of simulation parameters may be found in Table S1. We note here that the simulated magnitude of Δk is slightly lower than what may be expected based on the poling period and geometry of the device. Similarly, the d_{eff} used is slightly smaller than what has been previously reported for PPLN. Both numbers were adjusted to optimize the match between simulation and experiment as non-idealities such as thin-film thickness variation, fabrication error, and imperfect poling can create uncertainty in these quantities.

The results of these numerical simulations are used to generate the results of Figs. 1b and 1c of the main text as well as the entirety of Fig. 2. Although very good agreement is observed between these simulations and our experiments, some discrepancies may be observed, which we investigate here. The first is that our theory and simulation have assumed a transform-limited 35-fs pulse as our input to the compression device. In reality, as mentioned in Section

Parameter	Description	Value	Units
A_{eff}	effective mode area	2.18	µm^2
n_ω	refractive index, fundamental	1.88034	
$n_{2\omega}$	refractive index, second harmonic	2.06409	
Δk	phase mismatch	-4	rad mm^{-1}
d_{eff}	effective nonlinearity	11	pm V^{-1}
α_ω	loss, fundamental	0.023	mm^{-1}
$\alpha_{2\omega}$	loss, second harmonic	0.023	mm^{-1}
$\Delta\beta'$	GVM	27	fs mm^{-1}
$\beta_\omega^{(2)}$	GVD, fundamental	9.22	fs^2 mm^{-1}
$\beta_{2\omega}^{(2)}$	GVD, second harmonic	141	fs^2 mm^{-1}

Table S1: Simulation parameters for the soliton compression.

1.1, some pre-chirp is accumulated on the pulse due to propagation through several optical elements, with the main contribution being anomalous chirp from the ND wheel used to tune the input pulse energy to the compression system. We estimate the total accumulated group delay dispersion, accounting for chirp from the ND wheel, beam expander, and waveguide section prior to compression to be -260 fs^2.

Simulation results including this pre-chirp are shown in Fig. S11. The chirped input pulse is given in Fig. S11a and is shown to be 46 fs, similar to the experimentally measured 50-fs input pulses. The primary consequence of this pre-chirp is to slow the rate of compression down compared to the transform-limited case of Fig. 2. In particular, we see that the pulse is compressed to 9 fs at 2.9 pJ, compared to 7 fs in the unchirped case (Fig. S11b). Instead, compression to 7 fs is observed at 3.7 pJ of input energy (Fig. S11c). A small secondary consequence of the pre-chirp is that more energy flows into the small secondary lobe on the

leading edge of the pulse. Corresponding spectra for the input and two output pulses are shown in Figs. S11d-f.

That said, this result does not fully explain why the compressed pulses have a slightly longer duration than predicted, as it suggests that we should achieve the ideal 7-fs pulses by merely increasing the input pulse energy. To investigate this further, we turn to a fuller model which includes the effects of higher-order dispersion. Modeling the higher-order dispersion requires the following modification of Eqs. S2a and S2b:

$$\frac{\partial A_\omega}{\partial z} = i\kappa A_{2\omega} A_\omega^* e^{-i\Delta k z} + \hat{D}_\omega A_\omega - \frac{\alpha_\omega}{2}, \tag{S38a}$$

$$\frac{\partial A_{2\omega}}{\partial z} = i\kappa A_\omega^2 e^{i\Delta k z} - \Delta\beta' \frac{\partial A_{2\omega}}{\partial t} + \hat{D}_{2\omega} A_{2\omega} - \frac{\alpha_{2\omega}}{2}, \tag{S38b}$$

where we have considered the dispersion operator $\hat{D}_j = \sum_{m=2}^{\infty} \left[\frac{(i)^{m+1} \beta_j^{(m)}}{m!} \right] \partial_t^m$. Here, $\beta_j^{(m)}$ refers to the mth dispersion order in the jth wave. In our simulations, we include all orders of higher-order dispersion by calculating the propagation constant from the frequency-dependent index of Fig. S2e and subtracting the first two terms of the Taylor expansions about the fundamental and second-harmonic frequencies for the respective equations.

The results of this full simulation are shown in Fig. S12 for an input pulse energy of 2.9 pJ, mirroring the on-chip input energy used in the experiment. As in Fig. S11, we use a chirped input pulse to better capture the behaviors of the experiment. The output pulse profiles at the fundamental and second harmonic are shown in Figs. S12a and S12c, respectively. The fundamental pulse measures 16 fs, and the second harmonic measures 14 fs, in better agreement with the experimentally measured pulse durations compared to the simulations including only quadratic dispersion. Corresponding spectra in Figs. S12e and S12g also exhibit very similar behavior to the experimentally reconstructed spectra.

The shortest pulses observed in simulation occur for energies closer to 5 pJ. Here, we show a

simulated example at 4.6 pJ of pump pulse energy. In this case, pulse widths of 11 fs (Fig. S12b) and 10 fs (Fig. S12d) at the fundamental and second harmonic are observed. Corresponding spectra are shown in Figs. S12f and S12h. Most interestingly, a more prominent dip with two features is observed in the fundamental spectrum. The pulse widths and spectral shape agree well with experimental traces taken at 5 pJ of pump pulse energy; however, as discussed in Section 1.3, a phase ambiguity in the FROG measurement at these power levels prohibit a deterministic retrieval.

To further study the agreement between our model and experiment, we examine the spectrum and output pulse profile as a function of the input energy. The results are shown in Fig. S13. Figure S13a shows the spectrum evolution as the pump energy is increased for our simplified model which includes only quadratic dispersion. We note that the pump energy (y-axis) is plotted on a logarithmic scale. By comparison, the spectrum for the full simulation is shown in Fig. S13d. Finally, the experimental spectrum evolution as measured by the OSA is shown in Fig. S13g. Extremely good agreement with experiment is observed with both of the simulations, but especially for the full model. In particular, the fundamental and second-harmonic spectral components begin to strongly overlap at a similar point, near 4 pJ. Additionally, significant spectral splitting is observed near the center of the fundamental wavelength at 2090 nm for powers greater than 5 pJ in all cases.

We additionally compare the temporal profiles between the simulations and measurement. The fundamental pulse as a function of input energy for the three cases is shown in Figs. S13b, S13e, and S13h, while the second harmonic is shown in Figs. S13c, S13f, and S13i. As our FROG retrieval does not provide absolute temporal positioning for the measured pulses, we instead use the simulation including the full dispersion as a reference and position the measured pulses according to the maximum of their cross-correlation with the simulation for the same input energy. Each row, corresponding to different input energies, has been independently nor-

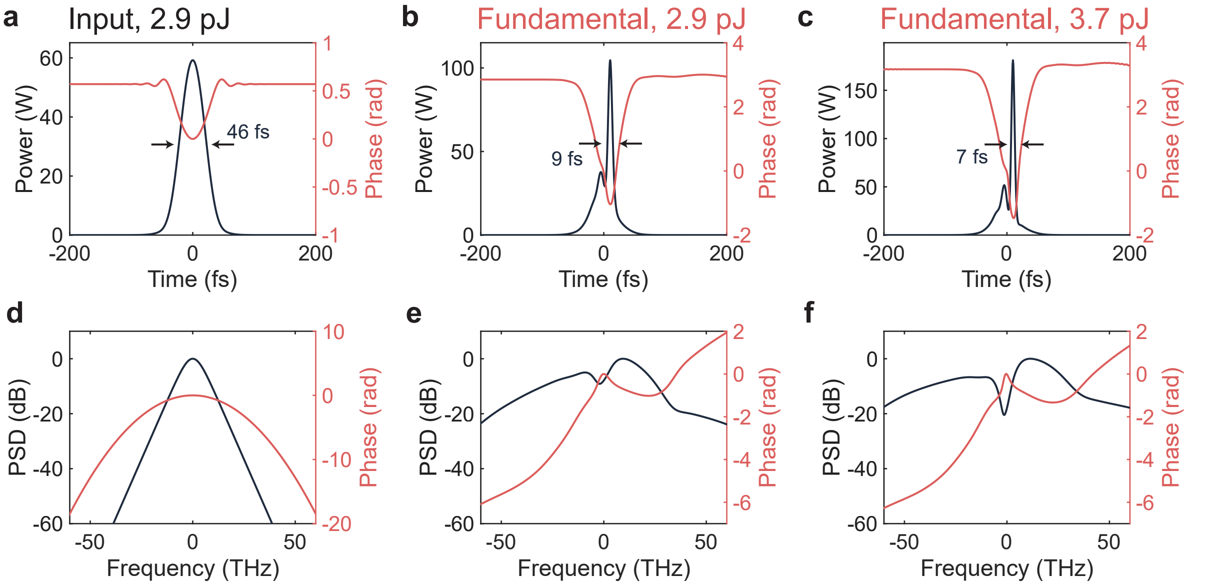

Figure S11: **Simulation results with pre-chirped input pulse**. **a**, Chirped input pulse used in simulation. **b**, Output at the fundamental for a 2.9-pJ input pulse. **c**, Output at the fundamental for a 3.7-pJ input pulse. **d**, Input spectrum. **e**, Output fundamental spectrum for a 2.9-pJ and **f**, 3.7-pJ input.

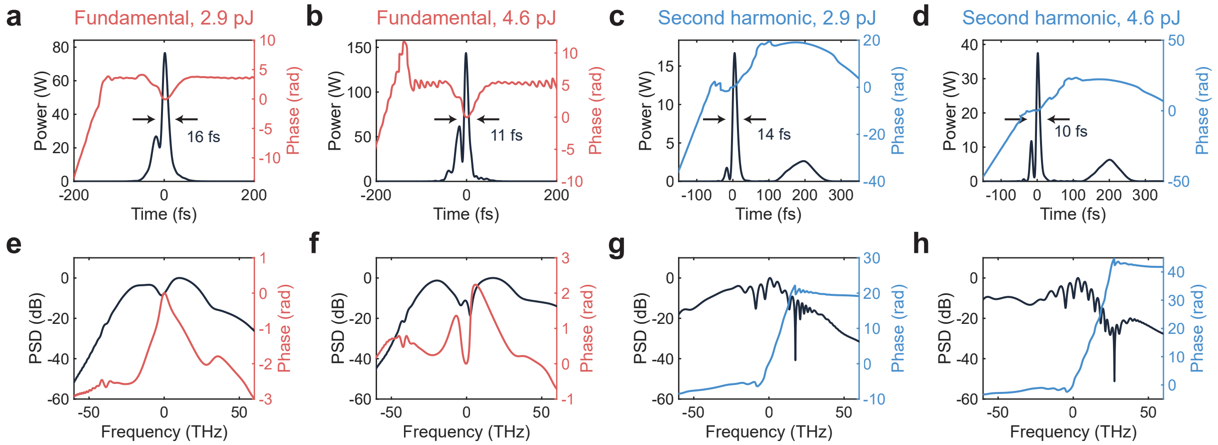

Figure S12: **Simulated output including higher-order dispersion**. **a**, Output fundamental pulse at 2.9 pJ and **b**, 4.6 pJ of pump pulse energy. **c**, Output second-harmonic pulse at 2.9 pJ and **d**, 4.6 pJ of pump pulse energy. The corresponding spectra are also plotted for the **e**, output fundamental at 2.9 pJ, **f**, output fundamental at 4.6 pJ, **g**, output second harmonic at 2.9 pJ, and **h**, output second harmonic at 4.6 pJ.

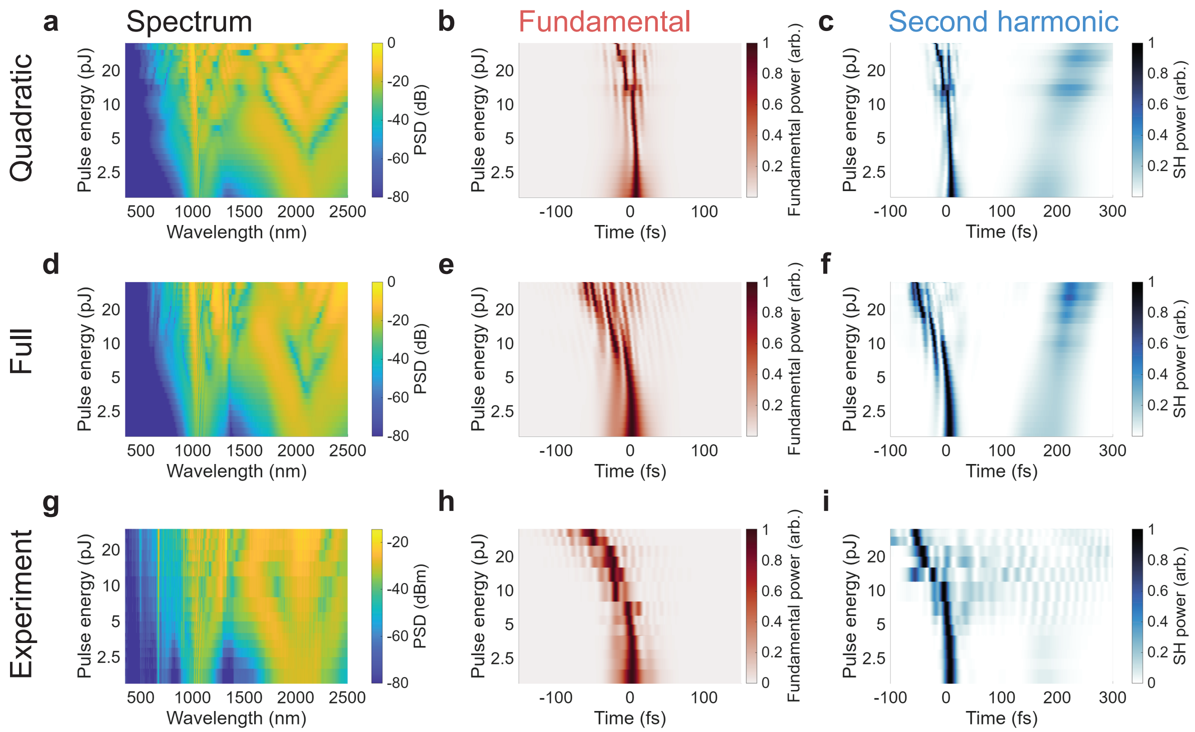

Figure S13: **Simulated vs. experimental energy-dependent outputs**. **a**, Simulated spectrum, **b**, fundamental pulse profile, and **c**, second-harmonic profile for various input energies, including only quadratic dispersion. For comparison, we show the **d**, spectrum, **e**, fundamental pulse, and **f**, second-harmonic pulse for the full simulation including higher-order dispersion. Finally, we show the experimentally-measured **g**, spectrum, **h**, fundamental pulse, and **i**, second-harmonic pulse for various input energies. The experimental pulse measurements are shifted according to the maximum of the cross-correlation with the full simulation at the same pump pulse energy.

malized to the pulse maximum. The measured pulses have further been interpolated onto the same time grid as the simulation to improve clarity.

For lower input energies, we see improved compression as the energy is increased, as expected. After the input energy exceeds about 5 pJ, however, the length of the device begins to exceed the optimum device length for compression, and the compression quality begins to degrade. In the case where only quadratic dispersion is included, the degradation is slower, with a single central feature being maintained until an input energy of around 12 pJ is reached. At this point, the device length is nearly double the calculated optimum for compression, and the pulse is seen to split into two distinct lobes. With the inclusion of higher-order dispersion, splitting is observed much sooner. This agrees well with our experiment, where the optimum compression is observed to occur with 3-5 pJ of pump pulse energy, and the quality quickly degrades for higher powers. In the experiment, however, the separate lobes predicted by the simulation are not well resolved at high powers. We attribute this primarily to the limitations of our measurement setup, as detailed in Section 1.3.

As a final point of comparison between experiment and simulation, and to illustrate the importance of operation in the soliton regime for such compression to occur, we characterize the behavior of a neighboring device which is nearly phase matched. The results are shown in Fig. S14. The measured spectrum as a function of input energy is shown in Fig. S14a, and the corresponding simulation including the full dispersion profile is shown in Fig. S14b. Although significant spectral broadening is seen to occur, we see many additional spectral features when compared to the soliton regime of Fig. S13. The simulated pulse profiles at the fundamental and second harmonic are shown in Figs. S14c and S14d, respectively. Here, significant pulse shortening is not observed, and the pulses are seen to split into many distinct lobes. This highlights the power of soliton pulse compression for achieving both broadband spectra and ultrashort pulses compared to other supercontinuum generation processes in quadratic media.

2.8 Extension to Longer Pump Pulses

While in our work we have begun with 35-fs input pulses, which are already quite short, the presented compression scheme may be readily extended to longer pump pulses. Here, we simulate the compression of an 80-fs pulse using the realistic parameters of our fabricated waveguide. Based on Eq. 6 of the main text, we calculate that for such a longer pulse, a 15.4-mm waveguide is required. Otherwise, the simulation parameters remain unchanged from those detailed in Section 2.6. We additionally consider an unchirped pump pulse.

Our simulation results are shown in Fig. S15. Due to the additional 9 mm of length in the waveguide, the pulse incurs an additional 1.8 dB of loss based on our simulation parameters, which must be compensated by pumping at a slightly higher power than the previously simulated 2.9-pJ inputs. The input 80-fs, 4.1-pJ pulse is shown in Fig. S15a. At the output of the waveguide, compressed 7-fs (S15b) and 8-fs (S15c) pulses are observed at the fundamental and second harmonic, similar to the outputs observed for the case of 35-fs pump pulses. The primary difference is the presence of a larger pedestal in the case of the longer pump pulse. Likewise, Fig. S15d shows the combined 4-fs pulse, indicating the possibility of using the compressed outputs for single-cycle pulse synthesis.

These results further demonstrate how the presented design framework may be flexibly used for the compression of a wide variety of input pulses. This flexibility ensures compatibility with emerging nanophotonic sources towards the development of integrated ultrafast systems.

2.9 Pulse Synthesis

As described in the main text, and theoretically outlined in Section 2.3, the phase relationship between the fundamental and second harmonic solitons enables tuning of the relative phase between the two harmonics during compression by simply modulating the phase of the input at the fundamental. In an experimental context, it is important also to be able to manipulate and

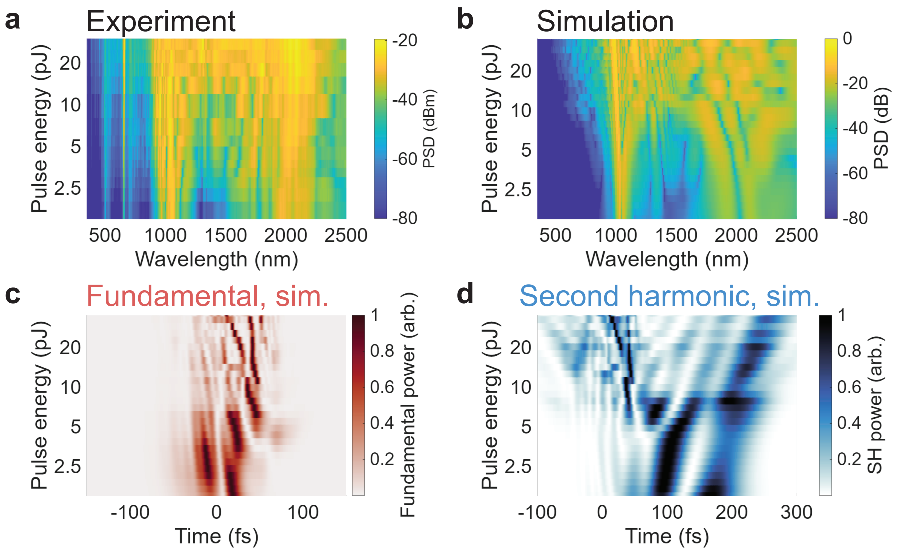

Figure S14: **Phase-matched supercontinuum generation**. **a**, Experimentally measured spectral broadening in the phase-matched regime. **b**, Simulation of phase-matched broadening using the full dispersion. **c**, Corresponding simulations of fundamental and **d**, second harmonic output pulses, demonstrating that temporal shortening is not observed in this regime.

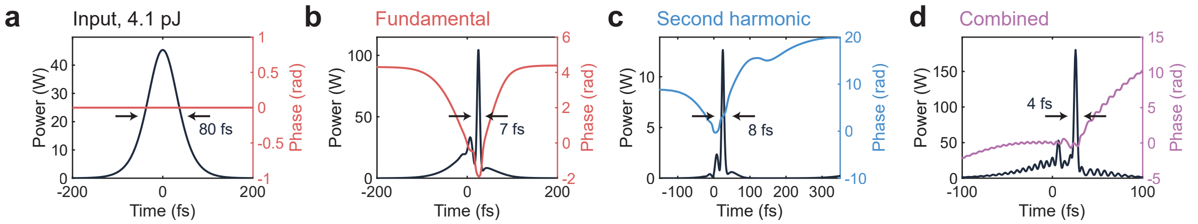

Figure S15: **Compression of 80-fs pulses**. **a**, Input 4.1-pJ, 80-fs pulse. **b**, Simulated temporal profile at the fundamental and **c**, corresponding second harmonic. **d**, Combined fundamental and second-harmonic pulses.

stabilize this relative phase to ensure a given desired pulse profile at the output. To do this, one may use the feedback loop shown in Fig. S16a.

Here, a carrier-envelope phase (CEP)-stable laser source is used to pump the nanophotonic chip consisting of the phase modulator and periodically poled region. For different relative phases, the two harmonics will interfere in the region of the spectrum where they overlap (near 1330 nm or 225 THz in the case of the present experiment). This interference can be seen in Fig. S4c. Thus, by monitoring a small portion of the output pulse on a photodetector via a 90:10 splitter and band-pass filtering around this spectral region, the relative phase of the two harmonics can be actively tracked. Side-of-fringe locking can then be achieved through a simple control loop.

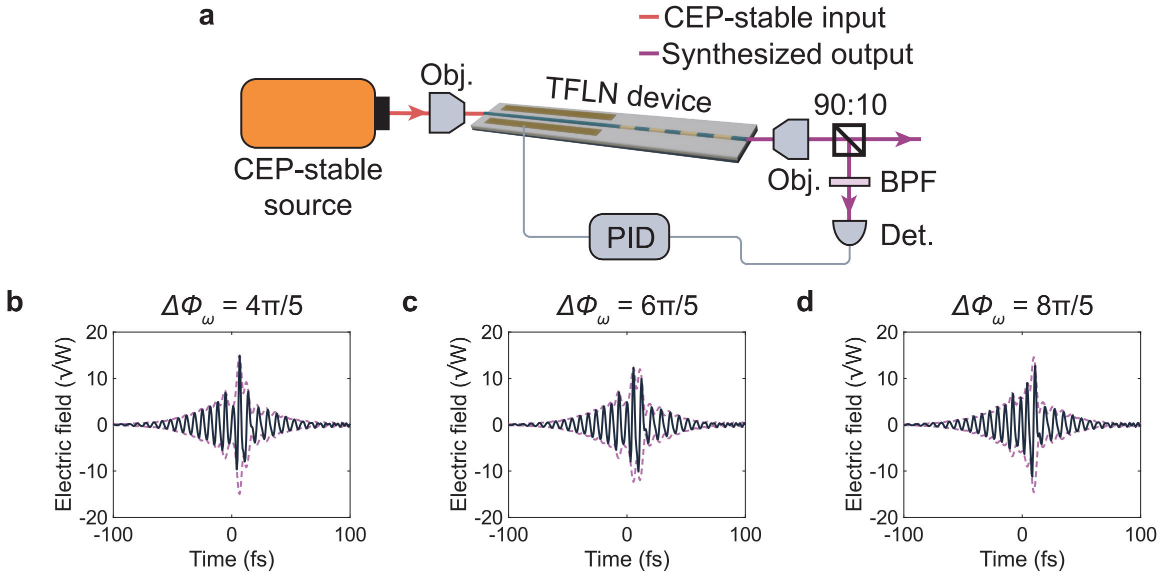

Figure S16: **Pulse synthesis**. **a**, Experimental setup for generating stabilized synthesized waveforms. **b-d**, Examples of synthesized fields for different values of the input envelope phase, $\Delta\phi_\omega$. CEP, carrier-envelope phase; Obj., objective; TFLN, thin-film lithium niobate; PID, proportional-integral-derivative; BPF, band-pass filter; Det., detector.

While Fig. 5b of the main text showed the temporal profile of the resulting pulse for several values of the input envelope phase shift, $\Delta\phi_\omega$, the synthesized field was shown only in the case

of $\Delta\phi_\omega = 0$, where the shortest pulse was achieved. Figures S16b-d show additional examples of electric field profiles that may be achieved for $\Delta\phi_\omega = 4\pi/5$, $6\pi/5$, and $8\pi/5$, respectively. While control is of course somewhat limited with only two harmonics to manipulate, we observe clear tuning of the relative strength of the two central cycles of the pulse.

References

1. Tamošauskas, G., Beresnevičius, G., Gadonas, D. & Dubietis, A. Transmittance and phase matching of BBO crystal in the 3- 5 μm range and its application for the characterization of mid-infrared laser pulses. *Optical Materials Express* **8,** 1410–1418 (2018).
2. Trebino, R. *Frequency-resolved optical gating: the measurement of ultrashort laser pulses* (Springer Science & Business Media, 2012).
3. Wyatt, A. *Frequency-resolved optical gating (FROG)* June 6, 2023. `https://www.mathworks.com/matlabcentral/fileexchange/16235-frequency-resolved-optical-gating-frog`.
4. Byrnes, S. *Frequency-resolved optical gating (FROG)* June 6, 2023. `https://www.mathworks.com/matlabcentral/fileexchange/34986-frequency-resolved-optical-gating-frog`.
5. Kane, D. J. Real-time measurement of ultrashort laser pulses using principal component generalized projections. *IEEE Journal of Selected Topics in Quantum Electronics* **4,** 278–284 (1998).
6. Kane, D. J. Recent progress toward real-time measurement of ultrashort laser pulses. *IEEE Journal of Quantum Electronics* **35,** 421–431 (1999).
7. Delong, K. W., Fittinghoff, D. N. & Trebino, R. Practical issues in ultrashort-laser-pulse measurement using frequency-resolved optical gating. *IEEE Journal of Quantum Electronics* **32,** 1253–1264 (1996).
8. Ratner, J., Steinmeyer, G., Wong, T. C., Bartels, R. & Trebino, R. Coherent artifact in modern pulse measurements. *Optics letters* **37,** 2874–2876 (2012).
9. Bourassin-Bouchet, C. & Couprie, M.-E. Partially coherent ultrafast spectrography. *Nature communications* **6,** 6465 (2015).
10. Keusters, D. *et al.* Relative-phase ambiguities in measurements of ultrashort pulses with well-separated multiple frequency components. *JOSA B* **20,** 2226–2237 (2003).
11. Guo, Q. *et al.* Ultrafast mode-locked laser in nanophotonic lithium niobate. *Science* **382,** 708–713 (2023).

12. Ledezma, L. *et al.* Intense optical parametric amplification in dispersion-engineered nanophotonic lithium niobate waveguides. *Optica* **9,** 303–308 (2022).
13. Jankowski, M., Mishra, J. & Fejer, M. Dispersion-engineered nanophotonics: a flexible tool for nonclassical light. *Journal of Physics: Photonics* **3,** 042005 (2021).
14. Boyd, R. W. *Nonlinear Optics* 3rd ed. (Academic Press, Cambridge, Massachusetts, 2008).
15. Kivshar, Y. S. & Agrawal, G. P. *Optical solitons: from fibers to photonic crystals* (Academic press, 2003).
16. Buryak, A. V., Di Trapani, P., Skryabin, D. V. & Trillo, S. Optical solitons due to quadratic nonlinearities: from basic physics to futuristic applications. *Physics Reports* **370,** 63–235 (2002).
17. Buryak, A. V. & Kivshar, Y. S. Spatial optical solitons governed by quadratic nonlinearity. *Optics letters* **19,** 1612–1614 (1994).
18. Sukhorukov, A. A. Approximate solutions and scaling transformations for quadratic solitons. *Physical Review E* **61,** 4530 (2000).
19. Karamzin, Y. N. & Sukhorukov, A. Nonlinear interaction of diffracted light beams in a medium with quadratic nonlinearity: mutual focusing of beams and limitation on the efficiency of optical frequency converters. *JETP Lett* **20,** 339–343 (1974).
20. Bache, M., Bang, O., Moses, J. & Wise, F. W. Nonlocal explanation of stationary and nonstationary regimes in cascaded soliton pulse compression. *Optics Letters* **32,** 2490–2492 (2007).
21. Boardman, A., Xie, K. & Sangarpaul, A. Stability of scalar spatial solitons in cascadable nonlinear media. *Physical Review A* **52,** 4099 (1995).
22. Bache, M., Moses, J. & Wise, F. Scaling laws for soliton pulse compression by cascaded quadratic nonlinearities. *JOSA B* **24,** 2752–2762 (2007).
23. Mollenauer, L. F., Stolen, R. H. & Gordon, J. P. Experimental observation of picosecond pulse narrowing and solitons in optical fibers. *Physical review letters* **45,** 1095 (1980).
